# Antibody–Drug Conjugates in Lung Cancer: Promise, Progress, and Persistent Challenges

**DOI:** 10.3390/cimb48090868

**Published:** 2026-08-26

**Authors:** Panagiotis Paliogiannis, Giorgia Fara, Angelo Zinellu, Alessandro Giuseppe Fois, Giuseppe Palmieri

**Affiliations:** 1 Department of Medicine, Surgery and Pharmacy, University of Sassari, Viale San Pietro 43, 07100 Sassari, Italy; agfois@uniss.it (A.G.F.); gpalmieri@uniss.it (G.P.); 2 Burn Center, University Hospital (AOU) of Sassari, Via Enrico De Nicola 39, 07100 Sassari, Italy; giorgia.fara@aouss.it; 3 Department of Biomedical Science, University of Sassari, Viale San Pietro 43, 07100 Sassari, Italy; azinellu@uniss.it; 4 Unit of Cancer Genetics, Institute of Genetic and Biomedical Research (IRGB), National Research Council (CNR), Traversa La Crucca n. 3, 07100 Sassari, Italy

**Keywords:** antibody-drug conjugates, lung cancer, NSCLC, SCLC, HER2, TROP2, resistance mechanisms, biomarkers, precision oncology

## Abstract

Lung cancer is currently the most frequently diagnosed malignancy worldwide, accounting for approximately 12.4% of all cancers and, despite major advances in molecularly targeted therapies and immunotherapy, represents the leading cause of cancer-related mortality. In recent years, antibody–drug conjugates (ADCs) have emerged as a novel therapeutic strategy, combining the specificity of monoclonal antibodies with the potent cytotoxic activity of highly active payloads to selectively target tumor cells while limiting systemic toxicity. This narrative review summarizes the current role of ADCs in lung cancer, with particular focus on their structural components, mechanisms of action, and the biological features that determine treatment efficacy. We discuss the rationale for targeting established and emerging antigens in non-small cell and small cell lung cancer, including HER2, TROP2, c-MET, HER3, CEACAM5, DLL3, and other promising targets currently under clinical investigation. The principal mechanisms of primary and acquired resistance are also reviewed, including antigen modulation, altered intracellular trafficking, lysosomal dysfunction, drug efflux, tumor microenvironment-mediated immune suppression, and intratumor heterogeneity. In addition, we provide an overview of the safety profile of ADCs, highlighting the most clinically relevant adverse events and their underlying biological mechanisms. We also examine the evolving landscape of predictive biomarkers beyond antigen expression, including genomic, transcriptomic, proteomic, and liquid biopsy-based approaches, together with emerging spatial and single-cell technologies that may improve patient selection. Finally, we discuss future directions in the field, including novel payloads, next-generation linker technologies, bispecific ADCs, combination strategies, and personalized ADC development. Overall, ADCs are rapidly reshaping the therapeutic landscape of lung cancer. Continued optimization of drug design, biomarker-driven patient selection, and a deeper understanding of resistance mechanisms will be essential to fully realize their clinical potential.

## 1. Introduction

Lung cancer was the most frequently diagnosed malignancy worldwide in 2024, accounting for approximately 2.63 million new cases (12.8% of all cancers), and remained the leading cause of cancer-related mortality, with an estimated 1.86 million deaths (19.1% of all cancer deaths) [1]. Recent epidemiological analyses have revealed a changing global burden of the disease, characterized by increasing incidence among women and in several low- and middle-income countries, while declining trends have been observed among men in most Western countries, largely reflecting historical changes in tobacco consumption [1,2]. Lung cancer is broadly classified into small-cell lung cancer (SCLC) and non-small-cell lung cancer (NSCLC), the latter accounting for approximately 85% of cases and including adenocarcinoma, squamous cell carcinoma (SCC), and large-cell carcinoma as its major histological subtypes [3]. Over the past several decades, adenocarcinoma has progressively replaced SCC as the predominant histological subtype worldwide and currently represents the largest proportion of cases in both men and women [4,5]. Despite advances in diagnosis and treatment, prognosis remains poor, with 5-year survival rates generally ranging from 10% to 20% worldwide [6,7,8].

Because lung cancer is often clinically silent in its early stages, most patients are diagnosed with locally advanced or metastatic disease and are therefore not eligible for surgical resection, which remains the treatment modality offering the greatest chance of long-term survival and cure [9]. The high proportion of patients presenting with unresectable disease has driven the development of increasingly effective systemic therapies, leading to a paradigm shift from conventional chemotherapy, historically associated with modest survival benefits, toward personalized treatment strategies guided by tumor biology. In this context, the advent of molecularly targeted therapies and immune checkpoint inhibitors (ICIs) has profoundly transformed the therapeutic landscape of lung cancer. The identification of actionable oncogenic drivers, including *EGFR*, *KRAS*, *BRAF*, *ALK*, *ROS1*, *MET*, *RET*, *NTRK* and *NRG1* alterations, has enabled the development of targeted agents capable of achieving durable clinical benefit in selected patient populations [10,11,12]. Similarly, ICIs have revolutionized the management of advanced disease by restoring antitumor immune responses and significantly improving survival outcomes, both as monotherapy and in combination with chemotherapy [13,14]. Nevertheless, many patients do not harbor actionable molecular alterations, fail to respond to immunotherapy, or develop acquired resistance, highlighting the persistent need for novel therapeutic approaches [15,16].

In this evolving therapeutic landscape, antibody–drug conjugates (ADCs) have emerged as a promising therapeutic strategy capable of addressing some of the limitations of both conventional chemotherapy and current precision oncology approaches. By combining the target specificity of monoclonal antibodies with the potent cytotoxic activity of chemotherapeutic payloads, ADCs enable selective drug delivery to tumor cells while potentially limiting systemic exposure [17,18]. Unlike molecularly targeted therapies, whose efficacy is generally restricted to tumors harboring specific genomic alterations, ADCs can exploit the expression of cell-surface antigens that are broadly represented across different molecular subgroups of lung cancer [19]. Encouraging clinical results achieved with several ADCs targeting HER2, TROP2, HER3, and other tumor-associated antigens have recently expanded the therapeutic options for both NSCLC and SCLC, positioning this drug class among the most promising innovations in thoracic oncology [19,20,21]. Given the rapidly evolving landscape of ADC development in lung cancer, this review aims to provide a comprehensive overview of their biological rationale and fundamental concepts, current clinical applications, emerging targets, mechanisms of resistance, and future perspectives.

## 2. ADC Fundamentals: Structure and Mechanisms of Action

Originally conceived as the embodiment of Paul Ehrlich’s “magic bullet” concept, ADCs were designed to achieve highly selective tumor targeting with minimal collateral toxicity; however, their development has been complex and characterized by progressive advances in antibody engineering, linker technology, and payload design [22,23]. Several technical and biological challenges inherent to ADCs, including their relatively high molecular weight, potential immunogenicity, and complex manufacturing processes, were recognized by the pioneers of the field and continue to represent major hurdles in their development [20,23]. Since the first Food and Drug Administration (FDA) approval of gemtuzumab ozogamicin in 2000 for the treatment of CD33-positive acute myeloid leukemia (AML), at least 21 additional ADCs have been approved worldwide for both hematological and solid malignancies, while more than 425 candidates are currently being evaluated in clinical trials [24,25].

**Figure 1:**
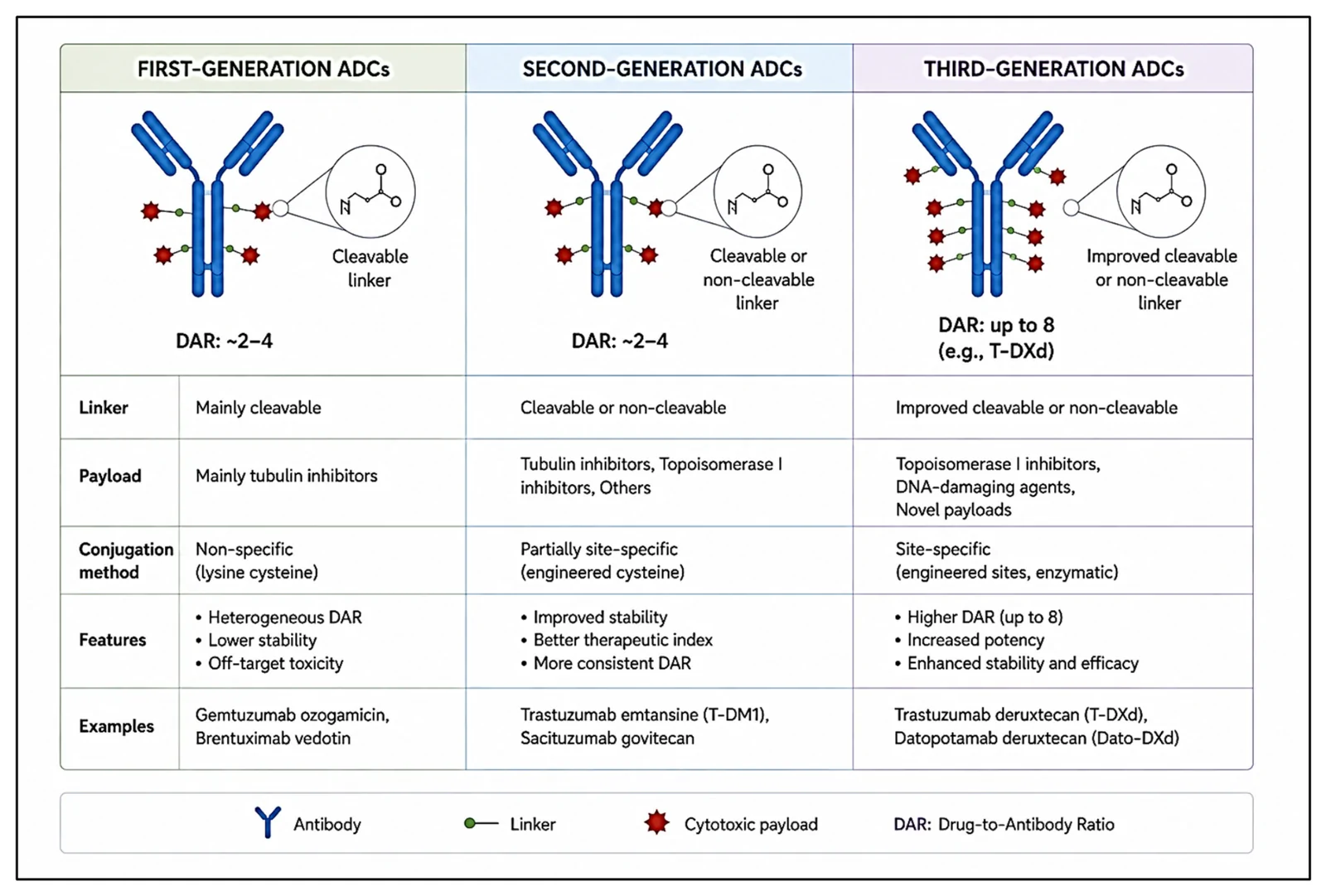
Evolution and main characteristics of successive generations of antibody–drug conjugates (ADCs), highlighting key advances in antibody engineering, linker technology, and payload design, and examples of the most commonly used medications available.

First-generation ADCs were limited by suboptimal linker stability, heterogeneous drug-to-antibody ratios (DARs), and the use of payloads with insufficient potency, resulting in premature drug release and a narrow therapeutic window [26]. Indeed, gemtuzumab ozogamicin, a representative example of this generation, was withdrawn from the market in 2010 because of significant safety concerns, particularly severe toxicity. Subsequent generations incorporated more stable conjugation strategies, improved linker chemistries, and highly potent cytotoxic agents, enhancing tumor-selective drug delivery while reducing systemic toxicity (Figure 1). More recently, the development of next-generation ADCs has been driven by advances in site-specific conjugation technologies, optimized DARs, improved cleavable linkers, and membrane-permeable payloads capable of eliciting a bystander effect. These innovations have expanded the scope of targeted drug delivery, and contributed to the emergence of novel conjugate platforms, including radionuclide drug conjugates (RDCs), small molecule–drug conjugates (SMDCs), virus-like drug conjugates (VDCs), antibody–oligonucleotide conjugates (AOCs), antibody–cell conjugates (ACCs), immune-stimulating antibody conjugates (ISACs), antibody fragment–drug conjugates (FDCs), antibody–degrader conjugates (ADeCs), and aptamer–drug conjugates (ApDCs) [20,27,28,29].

### 2.1. The Three Components of an ADC

**Figure 2:**
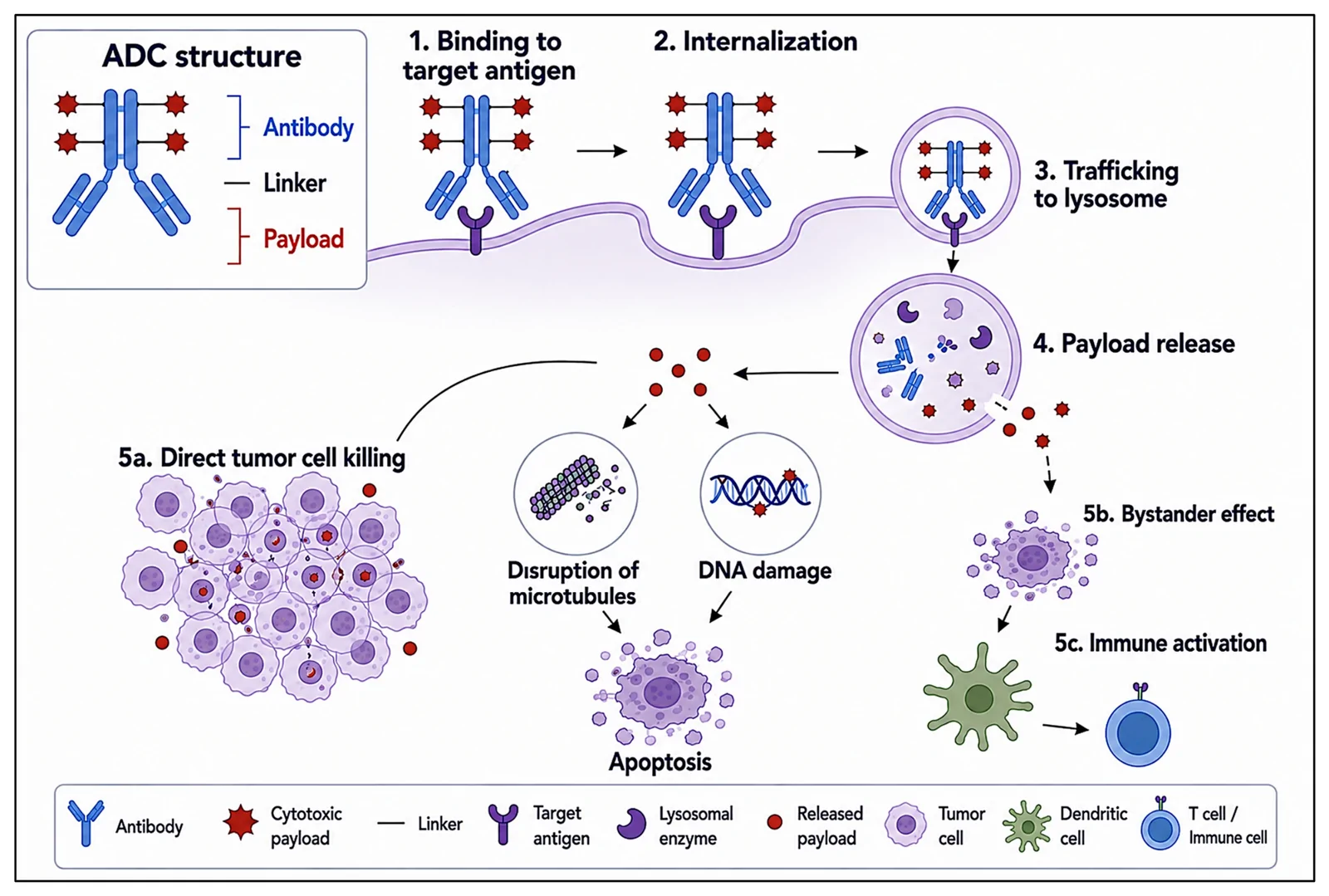
Conceptual overview of the main mechanisms of action of antibody–drug conjugates (ADCs). The figure schematically illustrates the major events following antigen binding, including internalization, intracellular trafficking, payload release, direct tumor cell killing, bystander effects, and immune activation. The representation is simplified and intended to highlight the principal biological mechanisms rather than provide a comprehensive depiction of all intracellular processes.

ADCs consist of three essential components: a monoclonal antibody, a chemical linker, and a cytotoxic payload (Figure 2). The antibody moiety is the targeting component of an ADC and mediates the selective recognition of antigens expressed on the surface of tumor cells, thereby directing the cytotoxic payload towards the intended target while minimizing its exposure to normal tissues [30,31].

The interaction between the antibody and its cognate antigen represents the first and most critical step in ADC activity, as efficient antigen binding promotes receptor-mediated internalization and subsequent intracellular delivery of the payload [31,32]. For this reason, monoclonal antibodies, which constitute the backbone of ADCs, must exhibit high target affinity and specificity, minimal off-target binding, and efficient tumor penetration and internalization to ensure therapeutic efficacy [33]. Moreover, the target antigen should be abundantly expressed on tumor cells while showing limited expression in normal tissues to maximize tumor selectivity and reduce on-target/off-tumor toxicity [30,31].

Most clinically approved ADCs employ humanized or fully human immunoglobulin G (IgG) antibodies, predominantly of the IgG1 subclass, owing to their long serum half-life, favorable stability, and well-characterized manufacturing properties [34,35]. In addition to serving as delivery vehicles, the antibody component may contribute directly to antitumor activity, depending on its characteristics, through blockade of receptor signaling pathways and engagement of immune effector mechanisms, including antibody-dependent cellular cytotoxicity (ADCC), antibody-dependent cellular phagocytosis (ADCP), and complement-dependent cytotoxicity (CDC) [35].

The linker represents a critical component of ADC design, as it covalently connects the monoclonal antibody to the cytotoxic payload and strongly influences the pharmacokinetic profile, efficacy, and safety of the conjugate. An ideal linker must exhibit sufficient stability in the systemic circulation to prevent premature payload release while ensuring efficient release once the ADC has reached the target cell. Consequently, linker chemistry plays a pivotal role in defining the therapeutic index of an ADC, balancing systemic stability with selective intracellular payload delivery [36,37]. Indeed, inadequate linker stability may result in premature drug release and off-target toxicity, whereas excessively stable linkers can impair payload release and compromise antitumor activity [37,38].

Based on their mechanism of payload release, linkers can be broadly classified as cleavable or non-cleavable [36,37,38]. Cleavable linkers respond to tumor-associated intracellular conditions, such as lysosomal protease activity, acidic pH, or reducing environments, to trigger selective payload release after ADC internalization. In contrast, non-cleavable linkers require complete proteolytic degradation of the antibody within lysosomes before the active drug can be released. While cleavable linkers may facilitate a bystander effect by enabling the diffusion of membrane-permeable payloads into neighboring tumor cells, non-cleavable linkers may provide greater plasma stability and limit premature systemic drug release [29,39]. Both strategies have demonstrated clinical success, and linker selection is now recognized as a key determinant of ADC pharmacology and overall therapeutic profile.

Finally, the payload represents the cytotoxic component of an ADC and is the primary determinant for its antitumor activity. Because only a limited fraction of the administered ADC reaches the tumor site and is successfully internalized, payloads must possess extremely high potency, typically several orders of magnitude greater than that of conventional chemotherapeutic agents [17]. Following intracellular release from the ADC, these compounds induce tumor cell death through a variety of mechanisms, including disruption of microtubule dynamics or induction of DNA damage [40]. Consequently, the physicochemical properties, potency, and mechanism of action of the payload are major determinants of ADC efficacy, safety, and bystander effect [41,42].

Most clinically approved ADCs employ payloads belonging to two major classes: microtubule inhibitors and DNA-damaging agents. Microtubule inhibitors include auristatins, such as monomethyl auristatin E (MMAE) and monomethyl auristatin F (MMAF), as well as maytansinoid derivatives including DM1 and DM4. These agents induce cell-cycle arrest and apoptosis through disruption of mitotic spindle function [31,43]. DNA-targeting payloads comprise highly potent compounds such as calicheamicins, duocarmycins, pyrrolobenzodiazepines, and topoisomerase I inhibitors, including deruxtecan (DXd) and SN-38, which induce DNA damage and subsequent tumor cell death [17]. More recently, topoisomerase I inhibitor-based payloads have emerged as an important and clinically successful platform in thoracic oncology, contributing to the clinical development of several next-generation ADCs, as we will discuss later [44].

### 2.2. Mechanisms of Action

A conceptual graphical summary of the mechanisms of action of ADCs is depicted in Figure 2. Following Fab-mediated binding of the ADC to its target antigen, the ADC-antigen complex undergoes receptor-mediated internalization and is subsequently trafficked through the endosomal-lysosomal pathway [45]. Within intracellular compartments, linker cleavage or proteolytic degradation of the antibody results in the release of the cytotoxic payload, which subsequently exerts its antitumor activity. Depending on the payload class, tumor cell death may occur through disruption of microtubule dynamics, leading to mitotic arrest and apoptosis, or through induction of DNA damage and replication stress, ultimately resulting in cell death, as mentioned above [40]. The efficiency of antigen internalization, intracellular trafficking, and payload release critically influences ADC activity and contributes to differences in efficacy among individual compounds.

Beyond direct target-cell killing, several ADCs can exert antitumor activity through a bystander effect [46]. Following intracellular release of the payload, membrane-permeable payloads may diffuse across cellular membranes and reach neighboring tumor cells regardless of their level of antigen expression [47]. This mechanism is particularly relevant in solid tumors, including lung cancer, where target expression is often heterogeneous both within and between tumor lesions. By extending cytotoxic activity beyond antigen-positive cells, the bystander effect may enhance tumor control and potentially mitigate one of the major limitations of highly selective targeted therapies. The magnitude of this effect is influenced by multiple factors, including payload membrane permeability, linker chemistry, and the physicochemical properties of the released drug [48,49].

In addition to their direct cytotoxic activity, ADCs may modulate the tumor microenvironment and promote antitumor immunity. The substantial tumor cell death induced by certain payloads can trigger immunogenic cell death, a process characterized by the release of tumor antigens and damage-associated molecular patterns (DAMPs), such as calreticulin, ATP, and high-mobility group box 1 (HMGB1) [49,50]. These signals can enhance antigen presentation by dendritic cells and promote the activation of tumor-specific T-cell responses. Moreover, as mentioned above, the antibody component itself may contribute to immune activation through Fc-mediated mechanisms, including ADCC and Fc-mediated phagocytosis [35,51]. The ability of ADCs to induce both direct tumor killing and immune-mediated effects has generated considerable interest in their combination with ICIs, an approach that is currently being investigated across multiple clinical trials.

## 3. ADC Targets in Lung Cancer

### 3.1. Non-Small Cell Lung Cancer (NSCLC)

#### 3.1.1. HER2

*HER2 (ERBB2)* is located on chromosome 17q12 and encodes a 185-kDa transmembrane receptor tyrosine kinase belonging to the ERBB family, which also includes EGFR, HER3, and HER4 [52]. Structurally, the receptor consists of an extracellular ligand-binding domain, a transmembrane region, and an intracellular kinase domain. Although HER2 has no known direct ligand, it acts as a preferred dimerization partner for other ERBB receptors, activating downstream signaling pathways such as PI3K/AKT and MAPK, which regulate cell proliferation, survival, and differentiation [53]. In NSCLC, HER2 alterations include activating mutations, particularly exon 20 insertions and, less frequently, gene amplification and rare gene fusions [10]. HER2 represents an attractive ADC target because it is expressed on the tumor cell surface, undergoes receptor-mediated internalization, and can be selectively exploited to deliver highly potent cytotoxic payloads to cancer cells [21]. For these reasons, HER2 has become one of the most extensively investigated ADC targets in NSCLC, with two HER2-directed ADCs currently recommended by the NCCN guidelines and several additional agents under clinical development [54].

#### 3.1.2. TROP2

TROP2 (trophoblast cell-surface antigen 2), encoded by the *TACSTD2* gene located on chromosome 1p32.1, is a 36-kDa transmembrane glycoprotein physiologically expressed in several epithelial tissues, where it contributes to cell proliferation, adhesion, regeneration, and calcium-mediated intracellular signaling [55]. Structurally, TROP2 consists of a large extracellular domain, a single transmembrane region, and a short intracellular tail containing phosphorylation sites involved in signal transduction. In NSCLC, TROP2 is frequently overexpressed at the protein level, particularly in adenocarcinoma, although activating genetic alterations such as mutations or gene fusions are uncommon and do not appear to play a major oncogenic role. Instead, increased TROP2 expression has been associated with tumor progression, epithelial–mesenchymal transition, metastatic potential, and poor clinical outcomes [56]. TROP2 is an attractive target for ADCs because of its abundant cell-surface expression, efficient receptor-mediated internalization, and differential expression in most normal tissues, enabling selective intracellular delivery of highly potent cytotoxic payloads [57]. Moreover, although a weak correlation between TROP2 expression and clinical activity has been reported for sacituzumab govitecan, this relationship may not be generalizable to all TROP2-directed ADCs and may depend on ADC-specific characteristics, including linker stability, payload properties, and bystander effects. These characteristics have established TROP2 as one of the most important ADC targets in NSCLC, with one FDA-approved agent and several next-generation compounds currently under clinical investigation [54].

#### 3.1.3. c-MET

c-MET, encoded by the proto-oncogene *MET* located on chromosome 7q31, is a transmembrane receptor tyrosine kinase whose physiological ligand is hepatocyte growth factor (HGF). The mature receptor consists of an extracellular α-chain and a transmembrane β-chain containing the intracellular tyrosine kinase domain [58]. Under normal conditions, HGF–MET signaling regulates embryonic development, tissue repair, cell proliferation, survival, motility, and epithelial regeneration through activation of the PI3K/AKT, RAS/MAPK, STAT3, and SRC signaling pathways [59]. In NSCLC, MET dysregulation can arise through several mechanisms, including MET exon 14 skipping mutations, gene amplification, protein overexpression, and, less commonly, gene fusions [60]. While MET exon 14 skipping and high-level MET amplification represent established oncogenic drivers, protein overexpression is considerably more frequent and may occur independently of underlying genomic alterations [61]. c-MET represents an attractive target for ADCs because it is highly expressed on the tumor cell surface, undergoes rapid receptor-mediated internalization following antibody binding, and displays relatively limited expression in normal tissues [62,63]. Importantly, the clinical activity of c-MET-directed ADCs appears to correlate more closely with protein overexpression than with genomic alterations, supporting the immunohistochemical (IHC) assessment of c-MET expression as an important biomarker for patient selection [64]. These characteristics have led to the clinical development of several c-MET-targeted ADCs, with telisotuzumab vedotin (Telizo-V) becoming the first agent approved for patients with c-MET-overexpressing NSCLC.

#### 3.1.4. HER3

HER3 (ERBB3), encoded by the *ERBB3* gene on chromosome 12q13, is a member of the ERBB family of receptor tyrosine kinases [65]. Structurally, HER3 comprises an extracellular ligand-binding domain, a transmembrane region, and an intracellular kinase domain that is catalytically impaired, rendering the receptor largely dependent on heterodimerization, particularly with HER2 or EGFR, for signal transduction [53]. Upon binding neuregulins (NRG1/NRG2), HER3 promotes activation of downstream pathways, most notably PI3K/AKT, as well as the MAPK pathway, thereby promoting cell proliferation, survival, differentiation, and resistance to apoptosis [20,21,23]. In NSCLC, HER3 is frequently overexpressed, whereas activating mutations, gene amplification, and gene fusions are uncommon and currently have not been established as major oncogenic drivers [66]. HER3 expression is particularly enriched in EGFR-mutant tumors, where it contributes to resistance to EGFR TKIs through compensatory signaling and reactivation of PI3K/AKT pathway activation [67]. These biological features make HER3 an attractive target for ADCs, as the receptor is abundantly expressed on the tumor cell surface and undergoes efficient antibody-mediated internalization despite its limited intrinsic kinase activity. Several HER3-directed ADCs, including patritumab deruxtecan, have demonstrated encouraging clinical activity in patients with EGFR-mutant NSCLC following progression on EGFR TKIs, establishing HER3 as one of the most promising emerging targets for next-generation ADC development in lung cancer [20,21,23].

#### 3.1.5. CEACAM5

CEACAM5 (carcinoembryonic antigen-related cell adhesion molecule 5), also known as carcinoembryonic antigen (CEA), is encoded by the *CEACAM5* gene located on chromosome 19q13.2 [68]. It is a glycosylphosphatidylinositol (GPI)-anchored cell-surface glycoprotein belonging to the immunoglobulin superfamily and is physiologically expressed on the apical surface of epithelial cells lining the gastrointestinal and respiratory tracts, where it contributes to cell adhesion, epithelial organization, and maintenance of tissue architecture [20,21,23]. In NSCLC, CEACAM5 is frequently overexpressed, particularly in lung adenocarcinoma, whereas activating mutations, gene amplifications, and gene fusions are uncommon and are not considered established oncogenic drivers [69]. Increased CEACAM5 expression has been associated with tumor progression, metastatic dissemination, and poorer clinical outcomes [69,70]. Its high and relatively homogeneous surface expression, together with efficient antibody binding and internalization, makes CEACAM5 an attractive target for ADC-based therapies. Several CEACAM5-directed ADCs are currently undergoing clinical evaluation, including tusamitamab ravtansine, which has demonstrated encouraging antitumor activity in patients with CEACAM5-positive NSCLC [20,21,23].

### 3.2. Small Cell Lung Cancer (SCLC)

Delta-like ligand 3 (DLL3) is currently one of the most extensively investigated ADC targets in SCLC. Encoded by the *DLL3* gene on chromosome 19q13, DLL3 is an inhibitory ligand of the Notch signaling pathway that is normally retained within the Golgi apparatus and expressed at very low levels in healthy adult tissues [71]. In contrast, DLL3 is aberrantly overexpressed on the surface of approximately 80–90% of SCLC cells, where it contributes to the maintenance of the neuroendocrine phenotype and tumor progression, making it an attractive therapeutic target with a potentially favorable tumor-to-normal tissue expression profile [71,72]. Beyond DLL3, several additional surface antigens are emerging as promising ADC targets in SCLC. B7-H3 (CD276), an immune checkpoint molecule frequently overexpressed in SCLC, has shown encouraging clinical activity with ifinatamab deruxtecan (I-DXd). Seizure-related 6 homolog (SEZ6), a neuronal membrane protein highly expressed in neuroendocrine tumors, is being investigated with ABBV-706, while other exploratory targets include TROP2, CD56 (NCAM1), and protein tyrosine kinase 7 (PTK7), although their clinical development remains at an earlier stage [73]. Collectively, these targets reflect the ongoing effort to expand the therapeutic options for SCLC, a disease that continues to have limited treatment options and poor long-term outcomes.

### 3.3. Other Emerging Targets

Beyond the most advanced targets, numerous additional tumor-associated antigens are currently being explored to further expand the therapeutic potential of ADCs in lung tumors. Among these, B7-H4 (VTCN1), an immune checkpoint molecule overexpressed in a subset of NSCLCs, has emerged as a promising candidate because of its restricted expression in normal tissues and favorable internalization properties [74]. PTK7, a pseudo-kinase involved in embryonic development and Wnt signaling, is frequently expressed in lung cancer and is being investigated with several next-generation ADCs [75]. Other emerging targets include AXL, a receptor tyrosine kinase implicated in epithelial–mesenchymal transition, metastasis, and resistance to targeted therapies [76]; ROR1, an oncofetal receptor associated with tumor stemness and poor prognosis [77]; LIV1 (SLC39A6), a zinc transporter expressed in epithelial malignancies [78]; and NECTIN4, a cell adhesion molecule already clinically validated as an ADC target in urothelial carcinoma and currently under evaluation in thoracic malignancies [79]. Additional antigens, including Claudin 18.2, EphA2, mesothelin, and MUC1, are also being evaluated in early-phase clinical development [80,81]. Continued identification of novel cell-surface targets, together with advances in their engineering and biomarker-driven patient selection, is expected to further broaden the clinical applications of ADCs. Table 1 summarizes the most relevant targets currently under investigation for ADC-based therapies.

## 4. Clinical Development and Current Recommendations for ADC Use in NSCLC

Currently, three ADCs have received FDA approval for the treatment of advanced NSCLC in selected molecularly defined patient populations (Table 2). These include (1) fam-trastuzumab deruxtecan (T-DXd), approved for patients with unresectable or metastatic NSCLC whose tumors harbor activating HER2 mutations; (2) datopotamab deruxtecan (Dato-DXd), approved for patients with EGFR-mutated NSCLC following prior treatment with an EGFR tyrosine kinase inhibitor and platinum-based chemotherapy; and (3) Telizo-V, approved for patients with previously treated non-squamous NSCLC exhibiting high c-MET protein overexpression [54]. In addition, ado-trastuzumab emtansine (T-DM1), although not FDA-approved for NSCLC, is currently included in the National Comprehensive Cancer Network (NCCN) guidelines as a potential subsequent-line treatment option for patients with HER2-mutant NSCLC [54].

### 4.1. HER2-Directed ADCs

T-DXd is a HER2-directed ADC composed of a humanized anti-HER2 monoclonal antibody linked to deruxtecan, a potent topoisomerase I inhibitor. It was the first ADC to demonstrate substantial clinical activity in HER2-mutant NSCLC and received accelerated FDA approval in 2022 for patients with previously treated advanced HER2-mutant disease. In the phase II DESTINY-Lung02 trial (NCT04644237), 102 patients received T-DXd at 5.4 mg/kg and 50 patients received T-DXd at 6.4 mg/kg intravenously every 3 weeks. T-DXd demonstrated a confirmed objective response rate (ORR) of 50.0% and 56.0% in the 5.4 mg/kg and 6.4 mg/kg groups, respectively, supporting its use as an established therapeutic option for patients with previously treated HER2-mutant NSCLC [82,83]. Notably, clinically meaningful intracranial activity was also observed, with responses reported in patients with baseline brain metastases; this finding was subsequently confirmed in large real-world cohorts [84]. These findings further established HER2 mutation as a therapeutically actionable target and highlighted the potential of ADCs to overcome some of the limitations associated with conventional HER2-directed therapies in lung cancer.

Beyond HER2-mutant disease, T-DXd has also demonstrated activity in tumors characterized by HER2 overexpression. In the phase II DESTINY-Lung01 trial (NCT03505710), patients with previously treated HER2-overexpressing NSCLC (IHC 2+ or 3+) received T-DXd at either 5.4 or 6.4 mg/kg intravenously every 3 weeks, achieving a confirmed ORR of 34.1% and 26.5%, respectively [85]. Among patients with HER2 IHC 3+ tumors, the ORR was 52.9% with the 5.4 mg/kg dose, supporting the clinical activity of T-DXd in HER2-overexpressing NSCLC. The safety profile of T-DXd is generally manageable, with the most frequent grade ≥ 3 adverse events being hematologic and gastrointestinal toxicities. However, interstitial lung disease (ILD) remains a distinctive and clinically relevant toxicity, reported in approximately 10–15% of treated patients across clinical studies, although severe events are uncommon. Based on the available evidence, current NCCN guidelines recommend T-DXd as a preferred subsequent therapy for both HER2-mutant and HER2-overexpressing NSCLC.

T-DXd is actively being studied as first-line treatment for advanced NSCLC harboring HER2 mutations or exhibiting HER2 overexpression; the phase III DESTINY-Lung04 trial (NCT05048797) is evaluating the ADC as monotherapy, and the phase III DESTINY-Lung06 study (NCT06899126) is evaluating T-DXd in combination with pembrolizumab and chemotherapy. Evaluation of T-DXd in the neoadjuvant setting in patients with HER2 mutations or HER2 overexpression has also begun in the phase II HERCULES trial (NCT07428044).

Although ado-trastuzumab emtansine (T-DM1) has not received FDA approval in NSCLC, it remains listed as a treatment option in NCCN guidelines for patients with HER2-mutant disease based on evidence of clinically meaningful activity from several phase II studies [54]. In a multicenter basket trial evaluating HER2-targeted therapies across different tumor types, T-DM1 achieved an ORR of 44% in patients with HER2-mutant lung cancer, with durable responses observed in a subset of patients [86]. Similar findings were subsequently reported in a phase II study conducted by the Japanese Lung Cancer Society, which demonstrated an objective response rate of 38.1% and a median progression-free survival of 2.8 months in previously treated HER2-mutant NSCLC [87]. Although its clinical role has been largely diminished following the emergence of T-DXd, which has demonstrated greater efficacy in HER2-mutant NSCLC, T-DM1 continues to be considered a potential therapeutic option in selected patients with HER2-mutant NSCLC.

### 4.2. TROP2-Directed ADCs

Dato-DXd is a TROP2-directed ADC that also delivers a DXd-based payload. In 2025, it received accelerated FDA approval for the treatment of adults with locally advanced or metastatic EGFR-mutated NSCLC who have received prior EGFR-directed therapy and platinum-based chemotherapy. Clinical development was supported by the phase III TROPION-Lung01 (NCT04656652) and phase II TROPION-Lung05 (NCT04484142) studies, in which Dato-DXd was administered at 6 mg/kg intravenously every 3 weeks, demonstrating meaningful antitumor activity in heavily pretreated patients [88]. Interestingly, TROP2 expression levels did not consistently correlate with treatment response, highlighting the limitations of antigen expression alone as a predictive biomarker, as we will discuss later in this review. In contrast, a pooled analysis of patients with EGFR-mutated NSCLC reported an ORR of 43%, supporting the clinical utility of Dato-DXd in this molecular subgroup [89]. In this pooled analysis, oral mucositis or stomatitis occurred in 69% of patients (grade ≥ 3, 9%). Ocular surface AEs were observed in 32% of patients, but grade 3 or higher AEs were rare (3%); none led to drug discontinuation. Drug-related ILD or pneumonitis, although uncommon (any grade, 4%; grade ≥ 3, <1%), was the adverse event most frequently leading to drug discontinuation. Based on these results, current NCCN guidelines list Dato-DXd among the preferred subsequent-line treatment options for EGFR-mutant NSCLC [54].

The clinical development of Dato-DXd continues to expand rapidly with first-line trials evaluating Dato-DXd in combination with immune checkpoint inhibitors in patients with EGFR wild-type NSCLC, including the phase III TROPION-Lung08 trial (NCT05215340) evaluating Dato-DXd plus pembrolizumab and the phase III TROPION-Lung10 trial (NCT06357533) investigating Dato-DXd plus rilvegostomig. Of particular interest is the recently published OptiTROP-Lung05 phase III trial which evaluated the TROP2-directed ADC sacituzumab tirumotecan (sac-TMT) combined with pembrolizumab versus pembrolizumab alone as first-line treatment for patients with PD-L1-positive advanced NSCLC lacking targetable genomic alterations [90]. The combination significantly improved progression-free survival (HR 0.35), with consistent benefit across PD-L1 subgroups, although this benefit was accompanied by a higher incidence of grade ≥ 3 adverse events. These findings suggest that sac-TMT plus pembrolizumab could reshape the first-line therapeutic standard for this patient population.

Beyond NSCLC, TROP2-directed ADCs are also being explored in small cell lung cancer, although the identification of reliable predictive biomarkers remains an unmet need.

### 4.3. c-MET ADCs

Teliso-V is a c-MET-directed ADC conjugated to the microtubule inhibitor MMAE as its cytotoxic payload. The agent received accelerated approval from the FDA in 2025 for adult patients with previously treated, locally advanced or metastatic non-squamous NSCLC characterized by high c-MET protein overexpression, defined as IHC 3+ staining in at least 50% of tumor cells. Clinical development was primarily supported by the multicohort phase II LUMINOSITY trial (NCT03539536), in which patients with EGFR-wild-type NSCLC and high levels of c-MET expression received Teliso-V at 1.9 mg/kg intravenously every 2 weeks, achieving an ORR of 35% [91]. Notably, antitumor activity was also observed in patients with intermediate c-MET expression, although with lower response rates (22.9%), suggesting that the optimal biomarker threshold for patient selection remains an area of ongoing investigation, as discussed later in this review.

The safety profile of Teliso-V is generally consistent with that generally associated with an MMAE-containing ADC. Peripheral sensory neuropathy represents the most common adverse event, occurring in approximately 30% of treated patients and leading to treatment discontinuation in approximately 10% of cases. Pulmonary toxicity appears to occur less frequently than that reported with deruxtecan-based ADCs, although pneumonitis remains a clinically relevant complication, occurring in 10.5% of patients, including grade ≥ 3 events in 2.9%. Based on the available evidence, current NCCN guidelines recommend Teliso-V as a subsequent-line treatment option for patients with EGFR-wild-type, non-squamous NSCLC exhibiting high c-MET expression [54]. Ongoing studies continue to refine its clinical positioning, including the phase III TeliMET NSCLC-01 trial (NCT04928846), which is evaluating the efficacy across different levels of c-MET expression, and a dedicated dose-optimization study comparing different schedules.

### 4.4. Other Targets and Ongoing Studies

The rapid expansion of the ADC landscape in thoracic oncology extends well beyond currently approved agents. Among the most advanced compounds in development is patritumab deruxtecan (HER3-DXd), a HER3-targeting ADC carrying a deruxtecan payload. Clinical development of HER3-DXd was supported by the phase II HERTHENA-Lung01 trial (NCT04619004), in which previously treated patients with EGFR-mutated NSCLC received HER3-DXd at 5.6 mg/kg intravenously every 3 weeks, achieving a confirmed ORR of 29.8%, a median PFS of 5.5 months, and a median OS of 11.9 months [92]; however, the subsequent phase III HERTHENA-Lung02 trial (NCT05338970) failed to demonstrate an overall survival benefit over platinum-based chemotherapy, highlighting the need for further investigation of HER3-DXd in molecularly defined patient populations [93].

Cofetuzumab pelidotin (Cofe-P) was investigated in the phase Ib M19-611 trial (NCT04189614), in which patients with PTK7-expressing recurrent NSCLC received Cofe-P at 2.8 mg/kg intravenously every 3 weeks, achieving an ORR of 18%, a median duration of response of 7.2 months, a median PFS of 5.5 months, and a median OS of 12.6 months; all patients experienced at least one adverse event, with alopecia (52%) and decreased neutrophil count (43%) being the most common, while grade ≥ 3 AEs occurred in 69% of patients, including grade ≥ 3 neutropenia in 25% [94]. Treatment-related AEs were reported in 94% of patients, with no treatment-related fatal events.

Novel ADC architectures are likewise emerging, such as izalontamab brengitecan (iza-bren; BL-B01D1), a bispecific ADC simultaneously targeting EGFR and HER3, which has shown encouraging activity in heavily pretreated patients with EGFR-mutant NSCLC. Bispecific ADCs are engineered to recognize two distinct tumor-associated antigens, potentially enabling enhanced tumor-cell binding and internalization while broadening target coverage and improving selectivity compared with conventional monospecific ADCs. Clinical development of iza-bren, a first-in-class EGFR/HER3 bispecific ADC, has been supported by phase I/II studies (NCT05194982 and NCT05880706), in which patients with previously treated EGFR-mutated NSCLC received iza-bren at the recommended phase II dose of 2.5 mg/kg intravenously on days 1 and 8 every 3 weeks, achieving a confirmed ORR of 48.8%, a median PFS of 6.9 months, and a median OS of 24.8 months [95]. Notably, iza-bren is now being evaluated in randomized phase II/III trials, underscoring its potential clinical relevance in EGFR-mutant NSCLC. AZD9592 is an emerging EGFR-directed ADC currently being investigated in a first-in-human phase I trial (NCT05647122), including in combination with osimertinib in EGFR-mutant NSCLC, although clinical efficacy data remain immature. A recent innovation in this context is represented by biparatopic HER2 ADCs, such as zanidatamab zovodotin (ZW49), which simultaneously binds two distinct HER2 epitopes and has shown preliminary clinical activity in HER2-expressing solid tumors in a first-in-human phase I trial (NCT03821233); however, further clinical data are needed to confirm these encouraging results.

Finally, novel payload strategies are also being explored beyond conventional cytotoxic agents. ISACs represent a particularly innovative approach, coupling tumor- or immune-cell-targeting antibodies with immunostimulatory payloads to induce localized immune activation [41]. XMT-2056, a HER2-directed ISAC carrying a STING agonist payload, is currently being evaluated in a phase I study (NCT05514717) that includes patients with HER2-positive NSCLC. Similarly, TAK-500 is an ISAC targeting CCR2-expressing myeloid cells and delivering a STING agonist to the tumor microenvironment; its phase I/II development includes patients with NSCLC (NCT05070247). These approaches may complement the direct cytotoxic activity of conventional ADCs by promoting innate and adaptive antitumor immune responses.

### 4.5. ADCs in Small Cell Lung Cancer

In SCLC, where substantial unmet therapeutic needs remain, several ADCs are generating considerable interest. Clinical development of ifinatamab deruxtecan (I-DXd), a B7-H3-directed ADC, has been supported by data from the phase II IDeate-Lung01 trial (NCT05280470) in previously treated extensive-stage SCLC, in which I-DXd was evaluated at 8 and 12 mg/kg intravenously every 3 weeks, with the 12 mg/kg dose selected for further clinical development; among patients receiving 12 mg/kg, I-DXd achieved an ORR of 48.2%, a median DOR of 5.3 months, a median PFS of 4.9 months, and a median OS of 10.3 months, while grade ≥ 3 treatment-related adverse events were reported in 36.5% of patients and treatment-related interstitial lung disease/pneumonitis was reported in 12.4% (grade ≥ 3, 4.4%) [96]. Other B7-H3-directed ADCs are also under active investigation. In parallel, ABBV-706, a SEZ6-directed ADC, has been investigated in the phase I first-in-human trial (NCT05599984), in which patients with relapsed/refractory SCLC received ABBV-706 at 1.8 or 2.5 mg/kg intravenously every 3 weeks, achieving an ORR of 52%; grade ≥ 3 treatment-related adverse events occurred in 61% of patients, with anemia (61%) and fatigue (38%) being the most common treatment-related adverse events, while the 1.8 mg/kg dose was selected as the recommended phase II dose based on the overall efficacy, safety, and pharmacokinetic profile [97]. Collectively, these agents illustrate the continuing diversification of ADC targets and formats, highlighting the potential of next-generation ADCs to expand therapeutic opportunities across multiple lung cancer subtypes.

## 5. Toxicities and Safety Considerations

### 5.1. General Principles and Class-Related Toxicities

The management of ADC-related toxicities remains a critical aspect of clinical care, particularly in advanced NSCLC, where underlying pulmonary impairment, cumulative treatment exposure, and reduced functional reserve may increase the risk and severity of adverse events. Despite their ability to selectively deliver cytotoxic agents to tumor cells, ADCs are associated with a broad spectrum of toxicities that largely reflect systemic exposure to their individual components, particularly their cytotoxic payloads, although the relative contribution of the antibody moiety to specific adverse events remains less well characterized [98]. Linker design also contributes significantly to the safety profile of ADCs. In particular, some enzyme-cleavable linkers may provide greater plasma stability and reduced premature payload release than acid-labile linkers, potentially resulting in a more favorable therapeutic index [99,100]. Moreover, the glycosylation pattern of the antibody may influence off-target uptake, as a high proportion of Fc agalactosylated glycans has been associated with increased accumulation in tissues enriched with myeloid, endothelial, and hepatic cells expressing mannose receptors [101].

As ADCs are increasingly incorporated into earlier treatment lines and curative-intent settings, including perioperative and adjuvant strategies, their long-term safety profile and the reversibility of treatment-related toxicities are becoming increasingly important considerations. Particular attention should be paid to the potential mutagenic effects of DNA-damaging payloads and to patient-related factors that may influence treatment tolerability. Variables such as age, sex, comorbidities, previous therapies, and individual genetic characteristics may support the development of personalized dosing strategies and toxicity management approaches [26].

Among the most common class-related toxicities are myelosuppression and gastrointestinal adverse events. Neutropenia, anemia, and thrombocytopenia are commonly observed across multiple ADC platforms and may reflect premature payload release, bystander effects, or uptake of ADC catabolites by normal proliferating cells. Likewise, nausea, vomiting, diarrhea, and anorexia are frequently reported, particularly with ADCs carrying topoisomerase I inhibitor payloads. The toxicity profile may vary according to the cytotoxic cargo. Microtubule inhibitors such as MMAE are commonly associated with peripheral neuropathy and ocular toxicity, whereas DM1-containing ADCs are frequently linked to thrombocytopenia [102]. In contrast, topoisomerase I inhibitor-based ADCs are more often associated with gastrointestinal and hematological adverse events. Additional toxicities may result from a complex interplay among target expression in normal tissues, linker stability, payload characteristics, and off-target drug exposure.

### 5.2. Organ-Specific Toxicities and Management

Among organ-specific toxicities, interstitial lung disease (ILD) has emerged as one of the most clinically relevant safety concerns, particularly for ADCs carrying DXd-based payloads (Table 3).

In DESTINY-Lung01, ILD was reported in 26% of patients treated with T-DXd, including two fatal cases [103]. Similarly, although generally associated with a favorable safety profile, Dato-DXd has been linked to stomatitis, pneumonitis, and grade ≥ 3 treatment-related adverse events in more than one-quarter of patients enrolled in TROPION-Lung05 [104]. A recent meta-analysis reported an overall incidence of pneumonitis of 5.9% for all-grade events and 0.7% for grade ≥ 3 events across ADCs, with T-DXd showing the highest risk (13.6% for all-grade and 2.2% for grade ≥ 3 events) [105]. Similarly, a dedicated meta-analysis of T-DXd monotherapy reported an overall ILD incidence of 15.4%, including fatal ILD events in 2.2% of treated patients [106]. As outlined above, in the LUMINOSITY trial, the most common treatment-related adverse event with Telizo-V was peripheral sensory neuropathy, occurring in 30.2% of patients and leading to treatment discontinuation in nearly 10% of cases [91]. Pulmonary toxicity was less common but remained clinically relevant, with pneumonitis reported in 10.5% of patients, including grade ≥ 3 events in 2.9%. Pneumonitis led to treatment discontinuation in 7.6% of treated patients.

Although the biological mechanisms underlying ADC-related ILD remain incompletely understood, current evidence suggests a multifactorial pathogenesis involving target-dependent and target-independent ADC uptake, extracellular diffusion of released payloads into pulmonary tissues, and exposure to circulating free payload generated by premature deconjugation [107]. These processes may promote the accumulation of cytotoxic compounds within alveolar macrophages and pulmonary parenchymal cells, ultimately leading to inflammatory lung injury and clinically significant ILD [108,109]. Clinical manifestations range from asymptomatic radiographic abnormalities to severe and potentially fatal pneumonitis. Consequently, early recognition is essential and requires a high index of suspicion in patients developing new respiratory symptoms or pulmonary infiltrates [110]. Current management strategies rely on prompt treatment interruption, early corticosteroid administration, and permanent discontinuation of the ADC in severe cases [110,111]. Increased awareness, routine radiological surveillance, and multidisciplinary management have substantially improved outcomes, making proactive monitoring a cornerstone of clinical practice [112,113].

Other clinically relevant toxicities require specific preventive and supportive measures. Oral mucositis can often be mitigated through appropriate oral care, including meticulous oral hygiene and steroid-containing mouth rinses, while symptomatic cases may require topical therapies and dose modifications. Ocular toxicities, such as dry eye and keratitis, are typically managed with prophylactic preservative-free artificial tears, avoidance of contact lenses, and prompt ophthalmological assessment when indicated. Infusion-related reactions are generally prevented through appropriate premedication and managed with temporary treatment interruption and supportive care [98].

## 6. Mechanisms of Resistance

**Figure 3:**
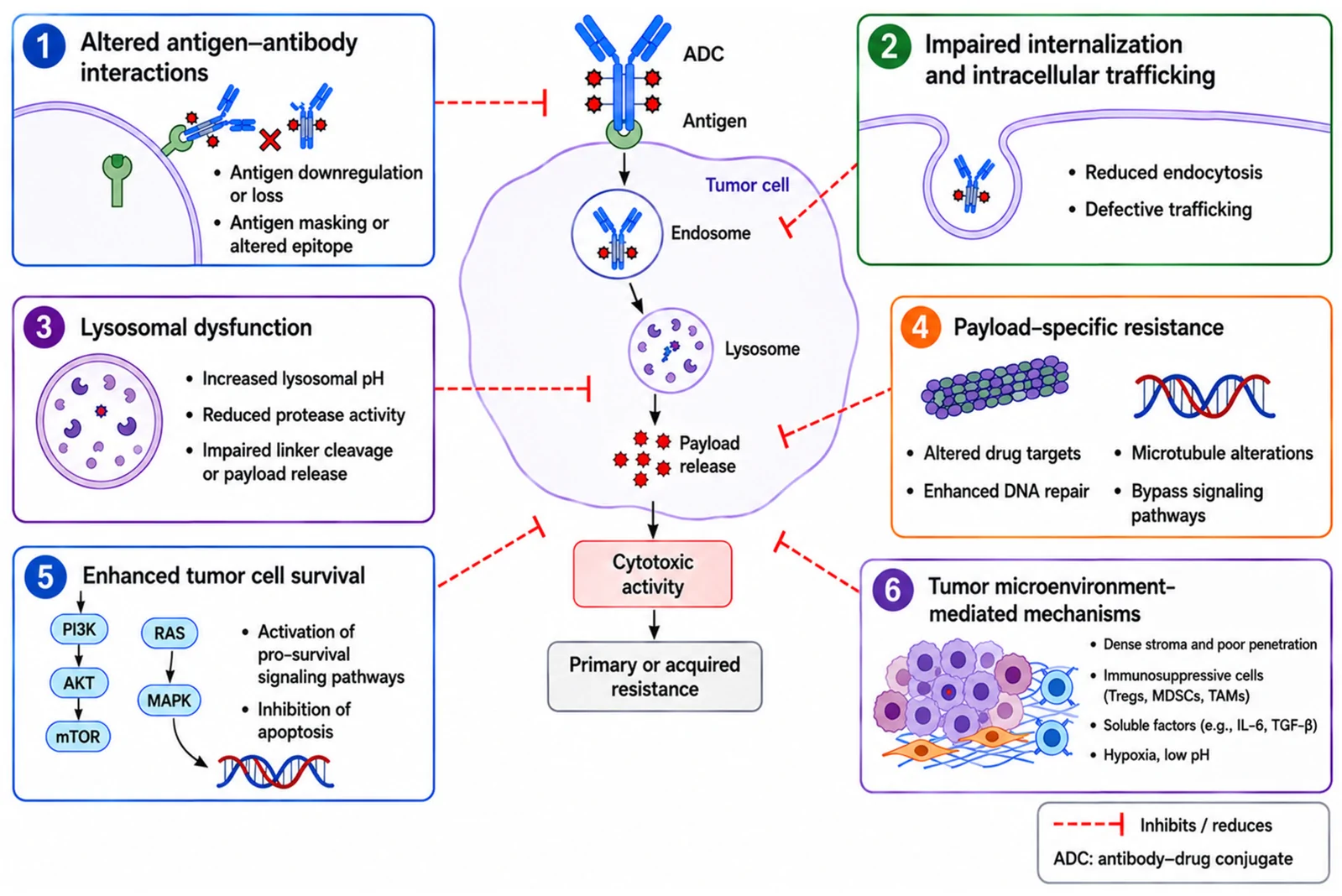
Major mechanisms of resistance to antibody–drug conjugates (ADCs). Schematic overview of the main mechanisms that may impair ADC uptake, intracellular processing, payload activity, or tumor-cell susceptibility, ultimately leading to primary or acquired treatment resistance. For completeness, Fc-mediated antibody–antigen interaction is also depicted, complementing the alternative Fab-mediated mechanism illustrated in Figure 2.

Resistance to ADCs is a multifactorial process that may arise through several distinct but often interconnected mechanisms, including alterations in antigen–antibody binding, impaired intracellular drug transport, lysosomal dysfunction, increased drug efflux, reduced payload sensitivity, activation of cancer cell survival pathways, and adaptive changes within the tumor microenvironment (Figure 3).

### 6.1. Antigen–Antibody Mediated Resistance

Resistance to ADCs can arise from multiple alterations affecting antibody–antigen interactions. Reduced or complete loss of antigen expression is among the most common mechanisms, as it impairs ADC binding, internalization, and intracellular payload delivery. This phenomenon has been described for several ADC targets, including HER2 and CD30, where resistant tumor cells exhibit lower antigen expression than their sensitive counterparts [114,115]. ADC efficacy may also be compromised by the presence of target circulating antigens or on tumor-derived exosomes, which can act as decoys and sequester the ADC before it reaches tumor cells [116,117]. In addition, structural alterations of the target receptor or masking of antibody-binding epitopes may reduce antigen recognition and limit ADC activity [118]. Finally, intratumoral heterogeneity represents a major obstacle to durable responses, as the coexistence of antigen-positive and antigen-negative cell populations enables the survival and expansion of resistant clones, ultimately leading to disease progression and treatment failure [119].

### 6.2. Resistance Due to Impaired Intracellular Drug Transport

Impaired intracellular trafficking represents another important mechanism of ADC resistance. Following antigen binding, ADCs must undergo efficient endocytosis and intracellular trafficking to reach lysosomes, where payload release takes place. Defects at any stage of this process may reduce intracellular drug delivery and compromise therapeutic efficacy. Resistance may arise from reduced internalization, enhanced receptor recycling, or alterations in endocytic pathways that divert ADCs away from lysosomal degradation. For example, reduced expression of proteins involved in receptor-mediated endocytosis can impair ADC uptake, whereas rapid recycling of target receptors back to the cell surface limits lysosomal trafficking and payload release [120]. This phenomenon is particularly relevant for HER2-targeted ADCs, as HER2 is preferentially recycled rather than degraded [121]. In addition, changes in the mechanism of internalization, including increased caveolae-mediated endocytosis, may redirect ADCs to intracellular compartments that are less favorable for lysosomal processing and payload release [122]. Collectively, these alterations reduce intracellular drug accumulation and represent a major obstacle to optimal ADC activity.

### 6.3. Resistance Due to Lysosomal Dysfunction

Lysosomal dysfunction represents a further mechanism of resistance to ADCs, as lysosomes are essential for intracellular payload processing and release of the active payload. Alterations in lysosomal biology, including impaired acidification and impaired payload export, can markedly reduce ADC efficacy. Non-cleavable ADCs are particularly dependent on efficient lysosomal proteolysis, which is required for degradation of the antibody component and subsequent release of the active payload catabolite [123]. Consequently, increased lysosomal pH, often resulting from reduced vacuolar H^+^-ATPase activity, may impair payload release and promote resistance [124]. In addition, lysosomes can act as intracellular reservoirs that sequester cytotoxic compounds, thereby limiting their access to intracellular targets [125]. Resistance may also arise from defects in lysosomal transport proteins involved in the export of ADC-derived catabolites into the cytoplasm. Reduced expression of proteins such as SLC46A3 has been associated with diminished sensitivity to several non-cleavable ADCs, highlighting the importance of efficient lysosomal trafficking for optimal ADC activity [126]. Together, these findings underscore the central role of lysosomal function in determining ADC responsiveness and suggest potential therapeutic strategies to overcome resistance.

### 6.4. Payload-Related Resistance

Resistance to ADCs may also develop at the level of the cytotoxic payload itself, through mechanisms commonly observed with conventional chemotherapy agents. Tumor cells can acquire reduced sensitivity to payload-induced cytotoxicity through alterations in the molecular targets of these agents, including microtubules and topoisomerase I. For microtubule-targeting payloads, changes in tubulin isoform expression or post-translational modifications may impair drug binding and diminish antitumor activity [127]. Likewise, resistance to topoisomerase I inhibitor-based payloads can arise through mutations affecting the target enzyme, altered intracellular localization of the target enzyme, enhanced DNA repair capacity, or defects in proteins involved in DNA damage responses [128,129]. In addition to intrinsic payload resistance, increased drug efflux represents a major mechanism of treatment failure. Overexpression of ATP-binding cassette transporters, particularly multidrug resistance proteins, can actively export released payloads from tumor cells, reducing intracellular drug accumulation below cytotoxic levels [130]. Although the contribution of efflux pumps may vary according to ADC design and payload characteristics, their upregulation has been consistently associated with resistance across multiple ADC platforms, highlighting their central role in limiting therapeutic efficacy [131].

### 6.5. Resistance Due to Enhanced Cancer Cell Survival

Enhanced cell survival represents another important mechanism of resistance to ADCs. Tumor cells may evade ADC-induced cytotoxicity through alterations in cell-cycle regulation or the activation of pro-survival signaling pathways. In particular, resistance to microtubule-targeting payloads has been linked to disruption of the spindle assembly checkpoint (SAC), a critical regulator of mitotic progression [132]. Overexpression of mitotic kinases such as polo-like kinase 1 (PLK1) or accelerated degradation of cyclin B1 can facilitate escape from mitotic arrest through a process known as mitotic slippage, allowing tumor cells to survive despite payload-induced microtubule disruption [133,134]. In parallel, dysregulation of apoptotic pathways may further reduce ADC sensitivity. Increased expression of anti-apoptotic proteins, including members of the BCL-2 family, and loss of pro-apoptotic mediators such as BAX and BAK can impair the execution of apoptotic programs triggered by ADC payloads [135]. These findings suggest that alterations in cell-cycle control and apoptosis not only contribute to ADC resistance but may also provide actionable therapeutic targets for combination strategies aimed at restoring treatment sensitivity.

### 6.6. Tumor Microenvironment Related Resistance

The tumor microenvironment (TME) represents an important source of both primary and acquired resistance to ADCs. Multiple stromal, molecular, and immune-related factors can limit ADC efficacy by promoting tumor cell survival and reducing sensitivity to payload-induced cytotoxicity. One mechanism involves the functional redundancy of receptor tyrosine kinase (RTK) signaling networks. Activation of alternative RTKs or increased production of growth factors within the TME can sustain downstream proliferative and survival pathways, including PI3K/AKT and MAPK signaling, thereby compensating for the effects of ADC-mediated target engagement and payload delivery [136,137]. In parallel, the immune microenvironment may profoundly influence treatment outcomes. Immunosuppressive cellular populations and inhibitory cytokines can attenuate antitumor immune responses, reducing the overall efficacy of ADC therapy [138]. Conversely, accumulating evidence indicates that several ADCs can induce immunogenic cell death, enhance antigen presentation, and promote adaptive immune responses, providing a strong biological rationale for combining ADCs with immune checkpoint inhibitors [139,140]. Together, these observations highlight the complex interplay between ADCs and the tumor microenvironment and support the development of combination strategies aimed at overcoming microenvironment-mediated resistance.

It should be acknowledged that much of the evidence discussed above derives from preclinical studies conducted across a variety of tumor types, particularly hematological malignancies and breast cancer, where ADCs have been used more extensively and for a longer period than in lung cancer. Importantly, emerging evidence from lung cancer is beginning to provide tumor-specific insights. In HER2-mutant NSCLC, acquired resistance to T-DXd has been associated with loss of SLFN11 and increased expression of the drug efflux transporter ABCC1/MRP1, resulting in reduced sensitivity to the deruxtecan payload. Secondary HER2 extracellular-domain alterations affecting the trastuzumab-binding site have also been identified, highlighting that resistance may arise at both the payload and target levels. Notably, HER2-mutant NSCLC models retaining HER2 signaling after T-DXd progression remained sensitive to HER2 tyrosine kinase inhibitors, suggesting that the mechanisms driving ADC resistance may differ from those conferring resistance to HER2-targeted kinase inhibition [141]. Similarly, emerging data from the ICARUS program in patients with NSCLC progressing on Dato-DXdsuggest that acquired resistance may involve broader biological changes, including activation of DNA repair pathways and suppression of immune-related pathways [142]. These findings underscore the need for further prospective studies specifically investigating ADC resistance in lung cancer, as mechanisms identified in other malignancies cannot necessarily be extrapolated across tumor types or ADC platforms.

## 7. Biomarkers: The Key to Precision ADC Therapy

### 7.1. Beyond Target Expression: Quantitative Assessment of ADC Targets

Target antigen expression remains the cornerstone biomarker for patient selection in ADC therapy; however, its predictive value remains limited. Clinical studies have shown that responses to HER2-directed ADCs may occur even in tumors with low or heterogeneous HER2 expression, while not all patients with high HER2 levels derive benefit [103,143]. Similarly, the relationship between TROP2 expression and response to TROP2-targeting ADCs remains inconsistent across studies [144]. These observations have highlighted the limitations of conventional binary biomarker assessment and prompted the development of more quantitative approaches. Digital pathology, standardized IHC scoring systems, and advanced imaging techniques are increasingly being explored to assess antigen density, distribution, and intratumoral heterogeneity [145,146]. From a clinical perspective, quantitative assessment of antigen expression and heterogeneity may improve patient selection by identifying patients more likely to achieve meaningful benefit from a given ADC. In particular, integrating antigen density and spatial distribution with standardized pathological assessment may help refine current eligibility criteria and potentially distinguish patients with low or heterogeneous target expression who may still benefit from ADC therapy.

### 7.2. Molecular Determinants of ADC Response and Resistance

Beyond target expression, several molecular features may influence ADC efficacy, as discussed above. Many mechanisms of primary or acquired resistance to ADCs are driven by specific molecular pathways and protein complexes, whose individual components may serve as potential predictive biomarkers of treatment response or resistance. Genomic alterations affecting payload sensitivity have emerged as particularly relevant biomarkers. For example, TOP1 expression and functional status may modulate responsiveness to topoisomerase I inhibitor-based ADCs, whereas deficiencies in DNA damage response genes such as BRCA1, BRCA2, and ATM may influence sensitivity to certain DNA-damaging payloads [147,148]. Similarly, high expression of SLFN11 has been associated with enhanced sensitivity to DNA-targeting agents, while upregulation of drug efflux transporters, including ABCB1 (MDR1), may contribute to treatment resistance [81]. Transcriptomic and proteomic analyses are further expanding our understanding of ADC biology by identifying pathways involved in receptor internalization, intracellular trafficking, lysosomal processing, and immune activation. Clinically, these molecular determinants could complement target expression in the prediction of treatment response and resistance. For example, assessment of payload sensitivity, DNA damage response pathways, drug efflux mechanisms, and other molecular features may help identify patients who are more likely to respond or develop resistance to specific ADC platforms.

### 7.3. Emerging Biomarker Technologies: Liquid Biopsy and Spatial Profiling

Novel technologies are expected to play an increasingly important role in ADC development and clinical implementation. Liquid biopsy approaches, including circulating tumor DNA (ctDNA), circulating tumor cells, and tumor-derived extracellular vesicles, offer a minimally invasive tool for molecular profiling when tissue biopsy is unfeasible or when available specimens are insufficient for comprehensive biomarker assessment. In addition, they enable longitudinal monitoring of tumor evolution, clonal dynamics, and the emergence of resistance mechanisms throughout treatment [149,150,151]. Liquid biopsy is now widely integrated into routine clinical practice for patients with NSCLC who are candidates for or receiving treatment with TKIs, and a variety of technologies and analytical platforms are available for clinical implementation [152,153,154]. The widespread adoption of these approaches has highlighted the need for methodological harmonization, leading to ongoing efforts to develop standardized protocols, quality assurance programs, and consensus recommendations [155]. From a clinical standpoint, the longitudinal nature of liquid biopsy makes it particularly attractive for ADC therapy, as serial ctDNA or circulating tumor cell analyses may allow early identification of molecular response, clonal evolution, and emerging resistance before radiographic progression becomes evident. This could facilitate dynamic treatment monitoring and potentially support earlier therapeutic adaptation.

In parallel, advances in spatial biology and single-cell technologies are providing unprecedented insights into tumor heterogeneity. Spatial transcriptomics enables the evaluation of gene expression within its tissue context, while single-cell sequencing can identify distinct cellular populations with differential antigen expression, resistance-associated phenotypes, and variable susceptibility to ADCs [156,157]. These approaches also allow detailed characterization of the tumor immune microenvironment, which may influence ADC activity and combination strategies [158]. Such approaches could help identify distinct cellular populations that may influence treatment response and guide the selection or sequencing of ADCs and combination therapies.

## 8. Future Perspectives and Persistent Challenges

The future development of ADCs in lung cancer will depend not only on the generation of increasingly potent constructs, but also on addressing several biological and clinical challenges that currently limit their broader application. Among the most important priorities are the identification of robust predictive biomarkers, optimization of the therapeutic window, development of novel payloads and ADC architectures, and rational integration of ADCs with other systemic therapies. Importantly, these areas are not equally mature, and their clinical translation will require different levels of evidence.

The development of novel payloads and linker technologies represents an important avenue for improving the therapeutic index of ADCs. Although topoisomerase I inhibitors and microtubule-disrupting agents currently represent the most successful payload platforms, next-generation ADCs are exploring alternative cytotoxic compounds, immune-stimulating molecules, and targeted inhibitors capable of modulating specific intracellular pathways [28,159]. However, increasing payload potency does not necessarily translate into greater clinical benefit, as systemic toxicity, uncontrolled payload release, bystander effects, and limited tumor penetration may offset gains in cytotoxic activity. Novel payload classes, including immune-stimulating agents, protein degraders, and other targeted intracellular compounds, remain largely investigational as we discussed earlier, and their clinical feasibility will depend on achieving sufficient tumor-selective delivery while limiting systemic and off-target toxicities. Similarly, advances in linker chemistry and site-specific conjugation aim to optimize payload stability during circulation and controlled intracellular release, while more homogeneous DARs may improve pharmacokinetic properties and therapeutic windows [160,161]. Thus, the next generation of ADCs should prioritize optimization of the overall therapeutic window rather than simply increasing payload potency.

Bispecific and other more sophisticated ADC architectures represent another promising strategy [162]. By simultaneously recognizing two distinct tumor-associated antigens, bispecific ADCs may improve tumor selectivity and internalization while reducing the impact of antigen heterogeneity or loss [25,163]. However, their increased molecular complexity may also introduce challenges related to manufacturing, pharmacokinetics, tissue penetration, and optimal target selection. Further clinical studies are therefore needed to establish whether the theoretical advantages of bispecific targeting translate into clinically meaningful improvements over conventional monospecific ADCs. Biparatopic approaches, such as HER2-directed ADCs recognizing two distinct epitopes of the same receptor, represent an additional strategy aimed at enhancing receptor engagement and internalization, although their clinical benefit remains to be established.

Among the most important priorities for the clinical development of ADCs is the identification of reliable predictive biomarkers. Although target expression remains the most widely used biomarker for patient selection, its ability to predict ADC activity varies substantially among individual agents. Target expression alone may be insufficient to capture the complex determinants of ADC sensitivity, which include antigen internalization, intracellular trafficking, linker stability, payload susceptibility, bystander activity, tumor heterogeneity, and mechanisms of resistance. Future biomarker strategies should therefore move beyond single-marker assessment toward integrated approaches combining tissue-based biomarkers, genomic alterations, transcriptomic profiles, and liquid biopsy-derived information. Advances in molecular profiling, spatial transcriptomics, and liquid biopsy technologies may facilitate this transition.

The optimal integration of ADCs with immunotherapy, targeted therapy, chemotherapy, and other systemic treatments represents another major unresolved question. Although both preclinical and clinical evidence provides a strong rationale for several combinations, additive efficacy must be balanced against overlapping toxicities, particularly hematologic, gastrointestinal, and pulmonary toxicities associated with selected ADC platforms [164]. Future clinical development should therefore prioritize biologically driven combinations and prospective biomarker selection rather than empirical treatment intensification. In HER2-mutant and EGFR-mutant NSCLC, the increasing availability of highly active targeted therapies further emphasizes the need to define the optimal sequencing of ADCs and targeted agents. Whether ADCs should be administered sequentially, combined with targeted therapies, or reserved for specific resistance settings remains an important clinical question that will require prospective randomized evaluation [165,166].

Finally, the role of ADCs is expected to extend beyond the treatment of advanced disease. Ongoing clinical trials are evaluating these agents in earlier disease settings, including neoadjuvant, adjuvant, and consolidation strategies, where effective eradication of micrometastatic disease could potentially improve long-term disease control and cure rates [167,168]. However, moving ADCs into earlier disease settings will require careful assessment of their long-term safety, particularly given the potential for cumulative or delayed toxicities, and demonstration of a clinically meaningful benefit over established treatment strategies.

## 9. Conclusions

ADCs represent one of the most significant therapeutic innovations in lung cancer over the last decade, expanding the treatment landscape beyond conventional chemotherapy, targeted therapies, and immunotherapy. Although only a limited number of ADCs have currently entered routine clinical practice, an unprecedented level of research activity is rapidly reshaping the field. Ongoing efforts encompass the development of novel payloads and linker technologies, bispecific and multifunctional ADC platforms, innovative combination strategies with targeted agents and ICIs, the identification of predictive biomarkers of response and resistance, and the implementation of more effective approaches for toxicity prevention and management. In parallel, the repertoire of actionable tumor-associated antigens continues to expand, while advances in ADC engineering are progressively improving drug delivery, tumor selectivity, and therapeutic efficacy. Collectively, these developments are expected to further broaden the clinical applicability of ADCs and strengthen their role in the management of both NSCLC and SCLC.

## Figures and Tables

**Table 1:** Principal antibody–drug conjugate targets in lung cancer and their biological and clinical characteristics.

Target	Gene (Chrom.)	Physiological Function	Main Alterations in Lung Cancer	Rationale for ADC Development	Representative ADCs
HER2	*ERBB2* (17q12)	Member of the ERBB receptor tyrosine kinase family; regulates proliferation, survival and differentiation through PI3K/AKT and MAPK signalling	Exon 20 insertions (most common), amplification, rare fusions	High cell-surface expression, efficient internalization, established oncogenic driver	Fam-trastuzumab deruxtecan; ado-trastuzumab emtansine; trastuzumab duocarmazine
TROP2	*TACSTD2* (1p32.1)	Cell adhesion, epithelial regeneration and intracellular calcium signalling	Protein overexpression (especially adenocarcinoma); mutations uncommon	Broad surface expression, rapid internalization, limited normal tissue expression	Datopotamab deruxtecan; sacituzumab govitecan; SKB264
c-MET	*MET* (7q31)	HGF receptor controlling proliferation, migration, tissue repair and survival	MET exon 14 skipping, amplification, protein overexpression, rare fusions	Strong membrane expression with rapid receptor-mediated internalization; IHC expression predicts response	Telisotuzumab vedotin; ABBV-400
HER3	*ERBB3* (12q13)	ERBB family receptor activating PI3K/AKT after heterodimerization with HER2 or EGFR	Protein overexpression; rare mutations/amplification	Highly expressed in EGFR-mutant NSCLC and internalizes efficiently after antibody binding	Patritumab deruxtecan; BNT326/YL202; izalontamab brengitecan
CEACAM5	*CEACAM5* (19q13.2)	Cell adhesion and maintenance of epithelial architecture	Protein overexpression, particularly in adenocarcinoma	High and relatively homogeneous surface expression with efficient antibody binding	Tusamitamab ravtansine
DLL3 (SCLC)	*DLL3* (19q13)	Inhibitory ligand of the Notch pathway	Aberrant overexpression in ~80–90% of SCLC	Minimal normal tissue expression and high tumour specificity	Rovalpituzumab tesirine; ZL-1310
B7-H3	*CD276* (15q24.1)	Immune checkpoint molecule	Frequent overexpression in SCLC and NSCLC	Restricted normal tissue expression and efficient internalization	Ifinatamab deruxtecan
SEZ6	*SEZ6* (17q11.2)	Neuronal membrane protein	Highly expressed in neuroendocrine tumours including SCLC	High tumour specificity	ABBV-706
Other emerging targets	*AXL*, *PTK7*, *ROR1*, *LIV-1*, *NECTIN4*, *B7-H4*, *CLDN18*, EPHA2, *MSLN*, *MUC1*	Cell signalling, adhesion, EMT and tumour progression	Variable overexpression according to tumour subtype	Potential for targeted payload delivery in biomarker-selected patients	Multiple investigational ADCs

**Table 2:** Main characteristics of ADCs currently recommended for clinical use in NSCLC.

ADC	Target	Payload	Linker	DAR	FDA Approval	Indication
Fam-trastuzumab deruxtecan	HER2	Deruxtecan (topoisomerase I inhibitor)	Cleavable tetrapeptide linker	~8	Yes	Subsequent line in HER2-mutated
Datopotamab deruxtecan	TROP2	Deruxtecan (topoisomerase I inhibitor)	Cleavable tetrapeptide linker	~4	Yes	Subsequent line in EGFR-mutated after TKI anti-EGFR + platinum-based ChT
Telisotuzumab vedotin	c-MET	MMAE (microtubule inhibitor)	Cleavable valine-citrulline linker	~3.1	Yes	cMET ≥ 50% IHC 3+ and EGFR wild type
Ado-trastuzumab emtansine	HER2	DM1 (maytansinoid microtubule inhibitor)	Non-cleavable MCC linker	~3.5	No	Subsequent line in HER2-mutated.

ChT: chemotherapy; c-MET: Mesenchymal–Epithelial Transition Factor; DM1: maytansinoid derivative 1; HER2: Human Epidermal Growth Factor Receptor 2; IHC: immunohistochemistry; MMAE: monomethyl auristatin E; TKI: tyrosine kinase inhibitor; TROP2: Trophoblast Cell Surface Antigen 2.

**Table 3:** Incidence of adverse events in the main regulatory trials testing ADCs in NSCLC.

Trial–Phase-Line	Target	Drug and Setting	Any TRAE	AE G3–4	AE G5	Interruption
DESTINY Lung02 Trial (NCT04644237) Phase II ≥2nd line	HER2	TDXd 5.4 mg/kg HER2 positive NSCLC	Global: 96% Nausea 67% Fatigue 45% Neutropenia 43% ↓ Appetite 40% Anemia 37% Constipation 37% Vomiting 32% Leukopenia 29% Thromb/penia 28% Diarrhea 23% Alopecia 22% ↑ AST/ALT 22% ILD 13%	Global: 39% Neutropenia 19% Anemia 11% Fatigue 8% Thromb/penia 6% Leukopenia 5% Nausea 4% ↑ AST/ALT 3% Vomiting 3% ↓ Appetite 2% Diarrhea 1% ILD 1%	1% ILD	Global: 14%
TROPION Lung01 Trial (NCT04656652) Phase III ≥2nd line	TROP2	Dato-DXd vs. Docetaxel	Global: 88% Stomatitis 48% Nausea 34% Alopecia 32% ↓ Appetite 23% Asthenia 19% Anemia 15% Diarrhea 10% ILD 9% Neutropenia 5% Leukopenia 3%	Global: 76% Stomatitis 7% ILD 4% Anemia 4% Asthenia 3% Nausea 2% Neutropenia 1%	1% ILD or Pneumon.	Global: 8% Pneumon. 4% ILD 1%
LUMINOSITY Trial NCT03539536 Phase II ≥2nd line	c-MET	Telizotumab Vedozin All NSCLC population	Global: 81% Per. neuropathy 30% Per. edema 16% Fatigue 14% ↓ Appetite 11% ↑ AST/ALT 10–11% Pneumonitis 11% Hypo/nemia 11% Blurred vision 9% Keratitis 6%	Global: 28% Per. Neuropathy 7% Fatigue 2% ↑ ALT 4% Pneumon. 3% Blurred vision 1%	2% ILD	21% Per. Neuropathy ILD or Pneumon.

ALT: Alanine Aminotransferase; AST: Aspartate Aminotransferase; Dato-DXd: Dapotomab Deruxtecan; Hypo/nemia: hypoalbuminemia; ILD: Interstitial Lung Disease; NSCLC: Non-Small Cell Lung Cancer; Per.: peripheral; Pneumon.: pneumonitis; TDXd: Trastuzumab Deruxtecan; Thromb/penia: thrombocytopenia.

## Data Availability

No new data were created or analyzed in this study. Data sharing is not applicable to this article.

## Abbreviations

The following abbreviations are used in this manuscript:

ACCs	antibody–cell conjugates
ADCC	antibody-dependent cellular cytotoxicity
ADCP	antibody-dependent cellular phagocytosis
ADCs	antibody–drug conjugates
ADeCs	antibody–degrader conjugates
AEs	adverse events
AML	acute myeloid leukemia
AOCs	antibody–oligonucleotide conjugates
ApDCs	aptamer–drug conjugates
CDC	complement-dependent cytotoxicity
Cofe-P	cofetuzumab pelidotin
ctDNA	circulating tumour DNA
DAMPs	damage-associated molecular patterns
DARs	drug-to-antibody ratios
Dato-DXd	datopotamab deruxtecan
DM1	maytansinoid derivative 1
DM4	maytansinoid derivative 4
FDA	Food and Drug Administration
FDCs	antibody fragment–drug conjugates
HMGB1	high-mobility group box 1
ICIs	immune checkpoint inhibitors
I-DXd	ifinatamab deruxtecan
IHC	immunohistochemistry
ILD	interstitial lung disease
ISACs	immune-stimulating antibody conjugates
Iza-bren	izalontamab brengitecan
MMAE	monomethyl auristatin E
MMAF	monomethyl auristatin F
NSCLC	non-small cell lung cancer
PLK1	polo-like kinase 1
PTK7	protein tyrosine kinase 7
RDCs	radionuclide drug conjugates
RTK	receptor tyrosine kinase
SAC	spindle assembly checkpoint
sac-TMT	sacituzumab tirumotecan
SCC	squamous cell carcinoma
SCLC	small cell lung cancer
SEZ6	Seizure-related homolog 6
SMDCs	small molecule-drug conjugates
T-DM1	ado-trastuzumab emtansine
T-DXd	fam-trastuzumab deruxtecan
Teliso-V	Telisotuzumab vedotin
TKIs	tyrosine kinase inhibitors
TME	tumour microenvironment
VDCs	virus-like drug conjugates

## References

[1] Sung H., Filho A.M., Laversanne M., Ferlay J., Siegel R.L., Soerjomataram I., Jemal A., Bray F. (2026). Global cancer statistics 2024: GLOBOCAN estimates of incidence and mortality worldwide for 34 cancers in 186 countries. CA Cancer J. Clin..

[2] Huang J., Deng Y., Tin M.S., Lok V., Ngai C.H., Zhang L., Lucero-Prisno D.E., Xu W., Zheng Z.J., Elcarte E. (2022). Distribution, risk factors, and temporal trends for lung cancer incidence and mortality: A global analysis. Chest.

[3] Inamura K. (2017). Lung cancer: Understanding its molecular pathology and the 2015 WHO classification. Front. Oncol..

[4] Luo G., Zhang Y., Rumgay H., Morgan E., Langselius O., Vignat J., Colombet M., Bray F. (2025). Estimated worldwide variation and trends in incidence of lung cancer by histological subtype in 2022 and over time: A population-based study. Lancet Respir. Med..

[5] Barta J.A., Powell C.A., Wisnivesky J.P. (2019). Global epidemiology of lung cancer. Ann. Glob. Health.

[6] Paliogiannis P., Colombino M., Sini M.C., Manca A., Casula M., Palomba G., Pisano M., Doneddu V., Zinellu A., Santeufemia D. (2022). Global prognostic impact of driver genetic alterations in patients with lung adenocarcinoma: A real-life study. BMC Pulm. Med..

[7] Bi J.H., Tuo J.Y., Xiao Y.X., Tang D.D., Zhou X.H., Jiang Y.F., Ji X.W., Tan Y.T., Yuan H.Y., Xiang Y.B. (2024). Observed and relative survival trends of lung cancer: A systematic review of population-based cancer registration data. Thorac. Cancer.

[8] Paliogiannis P., Attene F., Cossu A., Budroni M., Cesaraccio R., Tanda F., Trignano M., Palmieri G. (2013). Lung cancer epidemiology in North Sardinia, Italy. Multidiscip. Respir. Med..

[9] Passiglia F., Bertolaccini L., Del Re M., Facchinetti F., Ferrara R., Franchina T., Malapelle U., Menis J., Passaro A., Pilotto S. (2020). Diagnosis and treatment of early and locally advanced non-small-cell lung cancer: The 2019 AIOM (Italian Association of Medical Oncology) clinical practice guidelines. Crit. Rev. Oncol. Hematol..

[10] Fois S.S., Paliogiannis P., Zinellu A., Fois A.G., Cossu A., Palmieri G. (2021). Molecular epidemiology of the main druggable genetic alterations in non-small cell lung cancer. Int. J. Mol. Sci..

[11] Colombino M., Palomba G., Casula M., Pisano M., Tore S., Serra R., Putzu C., Fadda G.M., Maestrale G.B., Casula S. (2025). KRAS mutations in non-small cell lung cancer: Translational aspects, current therapies and challenges for future research. Crit. Rev. Oncol. Hematol..

[12] Colombino M., Paliogiannis P., Cossu A., Santeufemia D.A., Sini M.C., Casula M., Palomba G., Manca A., Pisano M., Sardinian Lung Cancer (SLC) Study Group (2019). EGFR, KRAS, BRAF, ALK, and cMET genetic alterations in 1440 Sardinian patients with lung adenocarcinoma. BMC Pulm. Med..

[13] Capella M.P., Pang S.A., Magalhaes M.A., Esfahani K. (2024). A review of immunotherapy in non-small-cell lung cancer. Curr. Oncol..

[14] Putzu C., Canova S., Paliogiannis P., Lobrano R., Sala L., Cortinovis D.L., Colonese F. (2023). Duration of immunotherapy in non-small cell lung cancer survivors: A lifelong commitment?. Cancers.

[15] Xiang Y., Liu X., Wang Y., Zheng D., Meng Q., Jiang L., Yang S., Zhang S., Zhang X., Liu Y. (2024). Mechanisms of resistance to targeted therapy and immunotherapy in non-small cell lung cancer: Promising strategies to overcoming challenges. Front. Immunol..

[16] Meyer M.L., Fitzgerald B.G., Paz-Ares L., Cappuzzo F., Jänne P.A., Peters S., Hirsch F.R. (2024). New promises and challenges in the treatment of advanced non-small-cell lung cancer. Lancet.

[17] Beck A., Goetsch L., Dumontet C., Corvaïa N. (2017). Strategies and challenges for the next generation of antibody-drug conjugates. Nat. Rev. Drug Discov..

[18] Ponziani S., Di Vittorio G., Pitari G., Cimini A.M., Ardini M., Gentile R., Iacobelli S., Sala G., Capone E., Flavell D.J. (2020). Antibody-drug conjugates: The new frontier of chemotherapy. Int. J. Mol. Sci..

[19] Cheng B., Gong L., Wang Z., Peng P., Xu K., Huang H., Wu P. (2026). Antibody-drug conjugates in oncology: Principles, clinical development, and future directions. MedComm.

[20] Zhou L., Lu Y., Liu W., Wang S., Wang L., Zheng P., Zi G., Liu H., Liu W., Wei S. (2024). Drug conjugates for the treatment of lung cancer: From drug discovery to clinical practice. Exp. Hematol. Oncol..

[21] Chen X., Zhou C. (2026). Antibody-drug conjugates in lung cancer: Current landscape and future perspectives. Curr. Opin. Oncol..

[22] Strebhardt K., Ullrich A. (2008). Paul Ehrlich’s magic bullet concept: 100 years of progress. Nat. Rev. Cancer.

[23] Colombo R., Tarantino P., Rich J.R., LoRusso P.M., de Vries E.G.E. (2024). The journey of antibody-drug conjugates: Lessons learned from 40 years of development. Cancer Discov..

[24] Zaytsev D., Girshova L., Ivanov V., Budaeva I., Motorin D., Badaev R., Mirolubova J., Grobovenko E., Chitanava T., Zaykova E. (2020). Rapid efficacy of gemtuzumab ozogamicin in refractory AML patients with pulmonary and kidney failure. Biology.

[25] Wang R., Hu B., Pan Z., Mo C., Zhao X., Liu G., Hou P., Cui Q., Xu Z., Wang W. (2025). Antibody-drug conjugates (ADCs): Current and future biopharmaceuticals. J. Hematol. Oncol..

[26] Dumontet C., Reichert J.M., Senter P.D., Lambert J.M., Beck A. (2023). Antibody-drug conjugates come of age in oncology. Nat. Rev. Drug Discov..

[27] Kim M.R., Lau J., Maffezzoli M., Banna G.L. (2026). Engineering the future of ADCs in non-small cell lung cancer. Oncol. Res..

[28] Tsuchikama K., Anami Y., Ha S.Y.Y., Yamazaki C.M. (2024). Exploring the next generation of antibody-drug conjugates. Nat. Rev. Clin. Oncol..

[29] Wu G., Yuan Z., Chen M., Tang X., Wang F., Zhang D. (2026). Antibody-drug conjugates (ADCs): A review of structural design, technological evolution, and future perspectives. Molecules.

[30] Parslow A.C., Parakh S., Lee F.T., Gan H.K., Scott A.M. (2016). Antibody-drug conjugates for cancer therapy. Biomedicines.

[31] Riccardi F., Dal Bo M., Macor P., Toffoli G. (2023). A comprehensive overview on antibody-drug conjugates: From the conceptualization to cancer therapy. Front. Pharmacol..

[32] Xu S. (2015). Internalization, trafficking, intracellular processing and actions of antibody-drug conjugates. Pharm. Res..

[33] Peters C., Brown S. (2015). Antibody-drug conjugates as novel anti-cancer chemotherapeutics. Biosci. Rep..

[34] Hoffmann R.M., Coumbe B.G.T., Josephs D.H., Mele S., Ilieva K.M., Cheung A., Tutt A.N., Spicer J.F., Thurston D.E., Crescioli S. (2017). Antibody structure and engineering considerations for the design and function of Antibody Drug Conjugates (ADCs). Oncoimmunology.

[35] Drago J.Z., Modi S., Chandarlapaty S. (2021). Unlocking the potential of antibody-drug conjugates for cancer therapy. Nat. Rev. Clin. Oncol..

[36] Jain N., Smith S.W., Ghone S., Tomczuk B. (2015). Current ADC linker chemistry. Pharm. Res..

[37] Su Z., Xiao D., Xie F., Liu L., Wang Y., Fan S., Zhou X., Li S. (2021). Antibody-drug conjugates: Recent advances in linker chemistry. Acta Pharm. Sin. B.

[38] Kostova V., Désos P., Starck J.B., Kotschy A. (2021). The chemistry behind ADCs. Pharmaceuticals.

[39] Bargh J.D., Isidro-Llobet A., Parker J.S., Spring D.R. (2019). Cleavable linkers in antibody-drug conjugates. Chem. Soc. Rev..

[40] Wang Z., Li H., Gou L., Li W., Wang Y. (2023). Antibody-drug conjugates: Recent advances in payloads. Acta Pharm. Sin. B.

[41] Xi M., Zhu J., Zhang F., Shen H., Chen J., Xiao Z., Huangfu Y., Wu C., Sun H., Xia G. (2024). Antibody-drug conjugates for targeted cancer therapy: Recent advances in potential payloads. Eur. J. Med. Chem..

[42] Li X., Liu J., Meng Y., Li J., Zhao J., Liu D., Zhang X. (2026). Frontiers in antibody-drug conjugates: Mechanisms, design innovations, and clinical applications in targeted cancer therapy. Pharmaceuticals.

[43] Widdison W.C., Wilhelm S.D., Cavanagh E.E., Whiteman K.R., Leece B.A., Kovtun Y., Goldmacher V.S., Xie H., Steeves R.M., Lutz R.J. (2006). Semisynthetic maytansine analogues for the targeted treatment of cancer. J. Med. Chem..

[44] Jang J.Y., Kim D., Lee N.K., Im E., Kim N.D. (2025). Antibody-drug conjugates powered by deruxtecan: Innovations and challenges in oncology. Int. J. Mol. Sci..

[45] Sutherland M.S., Sanderson R.J., Gordon K.A., Andreyka J., Cerveny C.G., Yu C., Lewis T.S., Meyer D.L., Zabinski R.F., Doronina S.O. (2006). Lysosomal trafficking and cysteine protease metabolism confer target-specific cytotoxicity by peptide-linked anti-CD30-auristatin conjugates. J. Biol. Chem..

[46] Khera E., Cilliers C., Smith M.D., Ganno M.L., Lai K.C., Keating T.A., Kopp A., Nessler I., Abu-Yousif A.O., Thurber G.M. (2021). Quantifying ADC bystander payload penetration with cellular resolution using pharmacodynamic mapping. Neoplasia.

[47] Wang Y., Cheng X., Li X., Chen W., Zhang X., Liu Y. (2025). Bystander effect in antibody-drug conjugates: Navigating the fine line in tumor heterogeneity. Crit. Rev. Oncol. Hematol..

[48] Giugliano F., Corti C., Tarantino P., Michelini F., Curigliano G. (2022). Bystander effect of antibody-drug conjugates: Fact or fiction?. Curr. Oncol. Rep..

[49] Forveille S., Zhao L., Sauvat A., Cerrato G., Leduc M., Doffe F., Pan Y., Liu P., Kroemer G., Kepp O. (2025). Patritumab deruxtecan induces immunogenic cell death. Oncoimmunology.

[50] Bauzon M., Drake P.M., Barfield R.M., Cornali B.M., Rupniewski I., Rabuka D. (2019). Maytansine-bearing antibody-drug conjugates induce in vitro hallmarks of immunogenic cell death selectively in antigen-positive target cells. Oncoimmunology.

[51] DiLillo D.J., Ravetch J.V. (2015). Fc-receptor interactions regulate both cytotoxic and immunomodulatory therapeutic antibody effector functions. Cancer Immunol. Res..

[52] Moasser M.M. (2007). The oncogene HER2: Its signaling and transforming functions and its role in human cancer pathogenesis. Oncogene.

[53] Yarden Y., Sliwkowski M.X. (2001). Untangling the ErbB signalling network. Nat. Rev. Mol. Cell Biol..

[54] NCCN Guidelines. https://www.nccn.org/guidelines/guidelines-detail?category=1&id=1450.

[55] Goldenberg D.M., Stein R., Sharkey R.M. (2018). The emergence of trophoblast cell-surface antigen 2 (TROP-2) as a novel cancer target. Oncotarget.

[56] Pak M.G., Shin D.H., Lee C.H., Lee M.K. (2012). Significance of EpCAM and TROP2 expression in non-small cell lung cancer. World J. Surg. Oncol..

[57] Lu H., Zhu M., Luo Y., Fan Q., Shang L., Wang N., Kong F. (2026). From biology to the clinic: Research evidence and therapeutic advances of TROP2-targeted antibody-drug conjugates in small cell lung cancer. Transl. Lung Cancer Res..

[58] Comoglio P.M., Trusolino L., Boccaccio C. (2018). Known and novel roles of the MET oncogene in cancer: A coherent approach to targeted therapy. Nat. Rev. Cancer.

[59] Organ S.L., Tsao M.S. (2011). An overview of the c-MET signaling pathway. Ther. Adv. Med. Oncol..

[60] Ye L., Wang W., Li H., Ji Y., Le X., Xu X. (2024). Targeting the MET gene: Unveiling therapeutic opportunities in immunotherapy within the tumor immune microenvironment of non-small cell lung cancer. Ther. Adv. Med. Oncol..

[61] Sacco J.J., Clague M.J. (2015). Dysregulation of the Met pathway in non-small cell lung cancer: Implications for drug targeting and resistance. Transl. Lung Cancer Res..

[62] Mer A.H., Mirzaei Y., Misamogooe F., Bagheri N., Bazyari A., Keshtkaran Z., Meyfour A., Shahedi A., Amirkhani Z., Jafari A. (2024). Progress of antibody-drug conjugates (ADCs) targeting c-Met in cancer therapy; insights from clinical and preclinical studies. Drug Deliv. Transl. Res..

[63] Yao H.P., Tong X.M., Hudson R., Wang M.H. (2020). MET and RON receptor tyrosine kinases in colorectal adenocarcinoma: Molecular features as drug targets and antibody-drug conjugates for therapy. J. Exp. Clin. Cancer Res..

[64] Tsao M.S., Sholl L., Shiller M., Illei P., Wistuba I.I., Beasley M.B., Schalper K.A., Simmons A., Ansell P., Beruti S. (2025). MET (c-Met) protein overexpression is an emerging protein biomarker in non-small cell lung cancer. npj Precis. Oncol..

[65] Uliano J., Corvaja C., Curigliano G., Tarantino P. (2023). Targeting HER3 for cancer treatment: A new horizon for an old target. ESMO Open.

[66] Karachaliou N., Lazzari C., Verlicchi A., Sosa A.E., Rosell R. (2017). HER3 as a therapeutic target in cancer. BioDrugs.

[67] Yonesaka K., Tanizaki J., Maenishi O., Haratani K., Kawakami H., Tanaka K., Hayashi H., Sakai K., Chiba Y., Tsuya A. (2022). HER3 augmentation via blockade of EGFR/AKT signaling enhances anticancer activity of HER3-targeting patritumab deruxtecan in EGFR-mutated non-small cell lung cancer. Clin. Cancer Res..

[68] Chan C.H., Stanners C.P. (2007). Recent advances in the tumour biology of the GPI-anchored carcinoembryonic antigen family members CEACAM5 and CEACAM6. Curr. Oncol..

[69] Lefebvre A.M., Adam J., Nicolazzi C., Larois C., Attenot F., Falda-Buscaiot F., Dib C., Masson N., Ternès N., Bauchet A.L. (2023). The search for therapeutic targets in lung cancer: Preclinical and human studies of carcinoembryonic antigen-related cell adhesion molecule 5 expression and its associated molecular landscape. Lung Cancer.

[70] Zhang X., Han X., Zuo P., Zhang X., Xu H. (2020). CEACAM5 stimulates the progression of non-small-cell lung cancer by promoting cell proliferation and migration. J. Int. Med. Res..

[71] Matsuo K., Taniguchi K., Hamamoto H., Inomata Y., Komura K., Tanaka T., Lee S.W., Uchiyama K. (2021). Delta-like canonical Notch ligand 3 as a potential therapeutic target in malignancies: A brief overview. Cancer Sci..

[72] Saunders L.R., Bankovich A.J., Anderson W.C., Aujay M.A., Bheddah S., Black K., Desai R., Escarpe P.A., Hampl J., Laysang A. (2015). A DLL3-targeted antibody-drug conjugate eradicates high-grade pulmonary neuroendocrine tumor-initiating cells in vivo. Sci. Transl. Med..

[73] Meng Y., Wang X., Yang J., Zhu M., Yu M., Li L., Liang Y., Kong F. (2024). Antibody-drug conjugates treatment of small cell lung cancer: Advances in clinical research. Discov. Oncol..

[74] Phipps M., Falchook G.S. (2025). B7 Homolog 4 (B7-H4)-directed agents in oncology clinical trials: A review. J. Immunother. Precis. Oncol..

[75] Maitland M.L., Sachdev J.C., Sharma M.R., Moreno V., Boni V., Kummar S., Stringer-Reasor E., Lakhani N., Moreau A.R., Xuan D. (2021). First-in-human study of PF-06647020 (Cofetuzumab Pelidotin), an antibody-drug conjugate targeting protein tyrosine kinase 7, in advanced solid tumors. Clin. Cancer Res..

[76] Gay C.M., Balaji K., Byers L.A. (2017). Giving AXL the axe: Targeting AXL in human malignancy. Br. J. Cancer.

[77] Tigu A.B., Munteanu R., Moldovan C., Rares D., Kegyes D., Tomai R., Moisoiu V., Ghiaur G., Tomuleasa C., Einsele H. (2024). Therapeutic advances in the targeting of ROR1 in hematological cancers. Cell Death Discov..

[78] Taylor K.M., Morgan H.E., Smart K., Zahari N.M., Pumford S., Ellis I.O., Robertson J.F., Nicholson R.I. (2007). The emerging role of the LIV-1 subfamily of zinc transporters in breast cancer. Mol. Med..

[79] Challita-Eid P.M., Satpayev D., Yang P., An Z., Morrison K., Shostak Y., Raitano A., Nadell R., Liu W., Lortie D.R. (2016). Enfortumab vedotin antibody-drug conjugate targeting nectin-4 is a highly potent therapeutic agent in multiple preclinical cancer models. Cancer Res..

[80] Nakada T., Sugihara K., Jikoh T., Abe Y., Agatsuma T. (2019). The latest research and development into the antibody-drug conjugate, [fam-] trastuzumab deruxtecan (DS-8201a), for HER2 cancer therapy. Chem. Pharm. Bull..

[81] Ascione L., Guidi L., Prakash A., Trapani D., LoRusso P., Lou E., Curigliano G. (2024). Unlocking the potential: Biomarkers of response to antibody-drug conjugates. Am. Soc. Clin. Oncol. Educ. Book.

[82] Jänne P.A., Goto Y., Kubo T., Ninomiya K., Kim S.W., Planchard D., Ahn M.J., Smit E., Johannes de Langen A., Pérol M. (2025). Final analysis results and patient-reported outcomes from DESTINY-Lung02-A dose-blinded, randomized, phase 2 study of trastuzumab deruxtecan in patients with HER2-mutant metastatic NSCLC. J. Thorac. Oncol..

[83] Jänne P.A., Planchard D., Goto K., Smit E.F., de Langen A.J., Goto Y., Ninomiya K., Kubo T., Pérol M., Felip E. (2025). Trastuzumab deruxtecan for ERBB2-mutant metastatic non-small cell lung cancer with or without brain metastases: A secondary analysis of randomized clinical trials. JAMA Netw. Open.

[84] Illini O., Hund A.C., Kopp H.G., Moskovitz M., Mountzios G., Brueckl W.M., Früh M., Leßmann M.E., Christopoulos P., Stenzinger A. (2026). Systemic and intracranial efficacy and safety of trastuzumab deruxtecan in patients with HER2-mutant NSCLC across treatment lines: Evidence from the TRACER/HERTras real-world cohort. J. Thorac. Oncol..

[85] Smit E.F., Felip E., Uprety D., Nagasaka M., Nakagawa K., Paz-Ares Rodríguez L., Pacheco J.M., Li B.T., Planchard D., Baik C. (2024). Trastuzumab deruxtecan in patients with metastatic non-small-cell lung cancer (DESTINY-Lung01): Primary results of the HER2-overexpressing cohorts from a single-arm, phase 2 trial. Lancet Oncol..

[86] Li B.T., Shen R., Buonocore D., Olah Z.T., Ni A., Ginsberg M.S., Ulaner G.A., Offin M., Feldman D., Hembrough T. (2018). Ado-trastuzumab emtansine for patients with HER2-mutant lung cancers: Results from a phase II basket trial. J. Clin. Oncol..

[87] Hotta K., Aoe K., Kozuki T., Ohashi K., Ninomiya K., Ichihara E., Kubo T., Ninomiya T., Chikamori K., Harada D. (2018). A phase II study of trastuzumab emtansine in HER2-positive non-small cell lung cancer. J. Thorac. Oncol..

[88] Ahn M.J., Tanaka K., Paz-Ares L., Cornelissen R., Girard N., Pons-Tostivint E., Vicente Baz D., Sugawara S., Cobo M., Pérol M. (2025). Datopotamab deruxtecan versus docetaxel for previously treated advanced or metastatic non-small cell lung cancer: The randomized, open-label phase III TROPION-Lung01 study. J. Clin. Oncol..

[89] Ahn M.J., Lisberg A., Goto Y., Sands J., Hong M.H., Paz-Ares L., Pons-Tostivint E., Pérol M., Felip E., Sugawara S. (2025). A pooled analysis of datopotamab deruxtecan in patients with EGFR-mutated NSCLC. J. Thorac. Oncol..

[90] Xiong A., Yao W., Zheng W., Yu Y., Chen P., Zhong H., Ge J., Wang H., Chen B., Wang H. (2026). Sacituzumab tirumotecan plus pembrolizumab versus pembrolizumab in PD-L1-positive advanced non-small-cell lung cancer (OptiTROP-Lung05): Interim analysis of a randomised, open-label, phase 3 trial. Lancet.

[91] Camidge D.R., Bar J., Horinouchi H., Goldman J., Moiseenko F., Filippova E., Cicin I., Ciuleanu T., Daaboul N., Liu C. (2024). Telisotuzumab vedotin monotherapy in patients with previously treated c-Met protein-overexpressing advanced nonsquamous EGFR-wildtype non-small cell lung cancer in the phase II LUMINOSITY trial. J. Clin. Oncol..

[92] Yu H.A., Goto Y., Hayashi H., Felip E., Chih-Hsin Yang J., Reck M., Yoh K., Lee S.H., Paz-Ares L., Besse B. (2023). HERTHENA-Lung01, a phase II trial of patritumab deruxtecan (HER3-DXd) in epidermal growth factor receptor-mutated non-small-cell lung cancer after epidermal growth factor receptor tyrosine kinase inhibitor therapy and platinum-based chemotherapy. J. Clin. Oncol..

[93] Mok T., Jänne P.A., Nishio M., Novello S., Reck M., Steuer C., Wu Y.L., Fougeray R., Fan P.D., Meng J. (2024). HERTHENA-Lung02: Phase III study of patritumab deruxtecan in advanced EGFR-mutated NSCLC after a third-generation EGFR TKI. Future Oncol..

[94] Cho B.C., Johnson M., Bar J., Schaefer E., Yoh K., Zer A., Moskovitz M., Lee S.H., Moreno V., de Miguel M. (2025). A phase 1b study of cofetuzumab pelidotin monotherapy in patients with PTK7-expressing recurrent non-small cell lung cancer. Lung Cancer.

[95] Hong S.D., Wang Y.S., Zhao H.Y., Zhao Y.Y., Wang Q.M., Ma Y.X., Li Y.S., Huang Y., Yang Y.P., Fu Z.M. (2026). Izalontamab brengitecan (Iza-bren; BL-B01D1), a first-in-class EGFR-HER3 bispecific antibody-drug conjugate, for patients with EGFR-mutated NSCLC: Pooled analysis of phase I and phase II trials. Ann. Oncol..

[96] Rudin C.M., Johnson M.L., Paz-Ares L., Nishio M., Hann C.L., Girard N., Rocha P., Hayashi H., Sakai T., Kim Y.J. (2026). Ifinatamab deruxtecan in patients with extensive-stage small cell lung cancer: Primary analysis of the phase II IDeate-Lung01 trial. J. Clin. Oncol..

[97] Byers L.A., Cho B.C., Cooper A.J., Chiang A.C., Han J.Y., Furqan M., Dowlati A., Morgensztern D., Papadopoulos K.P., Choudhury N.J. (2026). SEZ6-targeting antibody-drug conjugate ABBV-706 in advanced small cell lung cancer and solid tumors: A phase 1 trial. Nat. Med..

[98] Zhang Y.C., Luo W.C., Li J.T., Wu L., Chen Z.H., Zhou Q. (2026). The emerging role of antibody-drug conjugates in EGFR-mutant non-small cell lung cancer with acquired resistance to third-generation EGFR tyrosine kinase inhibitors. J. Control. Release.

[99] Cheng Y., Yuan X., Tian Q., Huang X., Chen Y., Pu Y., Long H., Xu M., Ji Y., Xie J. (2022). Preclinical profiles of SKB264, a novel anti-TROP2 antibody conjugated to topoisomerase inhibitor, demonstrated promising antitumor efficacy compared to IMMU-132. Front. Oncol..

[100] Ogitani Y., Aida T., Hagihara K., Yamaguchi J., Ishii C., Harada N., Soma M., Okamoto H., Oitate M., Arakawa S. (2016). DS-8201a, a novel HER2-targeting ADC with a novel DNA topoisomerase I inhibitor, demonstrates a promising antitumor efficacy with differentiation from T-DM1. Clin. Cancer Res..

[101] Gorovits B., Krinos-Fiorotti C. (2013). Proposed mechanism of off-target toxicity for antibody-drug conjugates driven by mannose receptor uptake. Cancer Immunol. Immunother..

[102] Tan H.N., Morcillo M.A., Lopez J., Minchom A., Sharp A., Paschalis A., Silva-Fortes G., Raobaikady B., Banerji U. (2025). Treatment-related adverse events of antibody drug-conjugates in clinical trials. J. Hematol. Oncol..

[103] Li B.T., Smit E.F., Goto Y., Nakagawa K., Udagawa H., Mazières J., Nagasaka M., Bazhenova L., Saltos A.N., Felip E. (2022). Trastuzumab deruxtecan in HER2-mutant non-small-cell lung cancer. N. Engl. J. Med..

[104] Sands J., Ahn M.J., Lisberg A., Cho B.C., Blumenschein G., Shum E., Pons Tostivint E., Goto Y., Yoh K., Heist R. (2025). Datopotamab deruxtecan in advanced or metastatic non-small cell lung cancer with actionable genomic alterations: Results from the phase II TROPION-Lung05 Study. J. Clin. Oncol..

[105] Zhu Z., Shen G., Li J., Qiu T., Fang Q., Zheng Y., Xin Y., Liu Z., Zhao F., Ren D. (2023). Incidence of antibody-drug conjugates-related pneumonitis in patients with solid tumors: A systematic review and meta-analysis. Crit. Rev. Oncol. Hematol..

[106] Powell C.A., Modi S., Iwata H., Takahashi S., Smit E.F., Siena S., Chang D.Y., Macpherson E., Qin A., Singh J. (2022). Pooled analysis of drug-related interstitial lung disease and/or pneumonitis in nine trastuzumab deruxtecan monotherapy studies. ESMO Open.

[107] Tarantino P., Modi S., Tolaney S.M., Cortés J., Hamilton E.P., Kim S.B., Toi M., Andrè F., Curigliano G. (2021). Interstitial lung disease induced by anti-ERBB2 antibody-drug conjugates: A review. JAMA Oncol..

[108] Koganemaru S., Fuchigami H., Morizono C., Shinohara H., Kuboki Y., Furuuchi K., Uenaka T., Doi T., Yasunaga M. (2025). Potential mechanisms of interstitial lung disease induced by antibody-drug conjugates based on quantitative analysis of drug distribution. Mol. Cancer Ther..

[109] Zhu J.Y., Jiang R.Y., Zhang H.P., Fang Z.R., Zhou H.H., Wei Q., Wang X. (2024). Advancements in research and clinical management of interstitial lung injury associated with ADC drugs administration in breast cancer. Discov. Oncol..

[110] Zhou C., Deng H., Yang Y., Wang F., Lin X., Liu M., Xie X., Luan T., Zhong N. (2025). Cancer therapy-related interstitial lung disease. Chin. Med. J..

[111] Heist R.S., Sands J., Bardia A., Shimizu T., Lisberg A., Krop I., Yamamoto N., Kogawa T., Al-Hashimi S., Fung S.S.M. (2024). Clinical management, monitoring, and prophylaxis of adverse events of special interest associated with datopotamab deruxtecan. Cancer Treat. Rev..

[112] Xu C., Chen Z., Xia Y., Shi Y., Fu P., Chen Y., Wang X., Zhang L., Li H., Chen W. (2024). Clinical best practices in interdisciplinary management of human epidermal growth factor receptor 2 antibody-drug conjugates-induced interstitial lung disease/pneumonitis: An expert consensus in China. Cancer.

[113] Yong W.P., Teo F.S., Teo L.L., Ng M.C., Tan T.J., Low S.Y., Wong K., Ang P., Choo S.P., Lee K.H. (2022). Clinical best practices in optimal monitoring, early diagnosis, and effective management of antibody-drug conjugate-induced interstitial lung disease or pneumonitis: A multidisciplinary team approach in Singapore. Expert. Opin. Drug Metab. Toxicol..

[114] Sabbaghi M., Gil-Gómez G., Guardia C., Servitja S., Arpí O., García-Alonso S., Menendez S., Arumi-Uria M., Serrano L., Salido M. (2017). Defective cyclin B1 induction in trastuzumab-emtansine (T-DM1) acquired resistance in HER2-positive breast cancer. Clin. Cancer Res..

[115] Chen R., Herrera A.F., Hou J., Chen L., Wu J., Guo Y., Synold T.W., Ngo V.N., Puverel S., Mei M. (2020). Inhibition of MDR1 overcomes resistance to brentuximab vedotin in Hodgkin lymphoma. Clin. Cancer Res..

[116] Aung T., Chapuy B., Vogel D., Wenzel D., Oppermann M., Lahmann M., Weinhage T., Menck K., Hupfeld T., Koch R. (2011). Exosomal evasion of humoral immunotherapy in aggressive B-cell lymphoma modulated by ATP-binding cassette transporter A3. Proc. Natl. Acad. Sci. USA.

[117] Ciravolo V., Huber V., Ghedini G.C., Venturelli E., Bianchi F., Campiglio M., Morelli D., Villa A., Della Mina P., Menard S. (2012). Potential role of HER2-overexpressing exosomes in countering trastuzumab-based therapy. J. Cell. Physiol..

[118] Scaltriti M., Rojo F., Ocaña A., Anido J., Guzman M., Cortes J., Di Cosimo S., Matias-Guiu X., Ramon y Cajal S., Arribas J. (2007). Expression of p95HER2, a truncated form of the HER2 receptor, and response to anti-HER2 therapies in breast cancer. J. Natl. Cancer Inst..

[119] Duhon A.A., McLean K. (2025). Navigating solid tumor heterogeneity: The promise and challenges of antibody-drug conjugates. Cancer Heterog. Plast..

[120] Baldassarre T., Truesdell P., Craig A.W. (2017). Endophilin A2 promotes HER2 internalization and sensitivity to trastuzumab-based therapy in HER2-positive breast cancers. Breast Cancer Res..

[121] Hunter F.W., Barker H.R., Lipert B., Rothé F., Gebhart G., Piccart-Gebhart M.J., Sotiriou C., Jamieson S.M.F. (2020). Mechanisms of resistance to trastuzumab emtansine (T-DM1) in HER2-positive breast cancer. Br. J. Cancer.

[122] Sung M., Tan X., Lu B., Golas J., Hosselet C., Wang F., Tylaska L., King L., Zhou D., Dushin R. (2018). Caveolae-mediated endocytosis as a novel mechanism of resistance to trastuzumab emtansine (T-DM1). Mol. Cancer Ther..

[123] DeVay R.M., Delaria K., Zhu G., Holz C., Foletti D., Sutton J., Bolton G., Dushin R., Bee C., Pons J. (2017). Improved lysosomal trafficking can modulate the potency of antibody drug conjugates. Bioconjug. Chem..

[124] Ríos-Luci C., García-Alonso S., Díaz-Rodríguez E., Nadal-Serrano M., Arribas J., Ocaña A., Pandiella A. (2017). Resistance to the antibody-drug conjugate T-DM1 is based in a reduction in lysosomal proteolytic activity. Cancer Res..

[125] Zhitomirsky B., Assaraf Y.G. (2016). Lysosomes as mediators of drug resistance in cancer. Drug Resist. Updat..

[126] Hamblett K.J., Jacob A.P., Gurgel J.L., Tometsko M.E., Rock B.M., Patel S.K., Milburn R.R., Siu S., Ragan S.P., Rock D.A. (2015). SLC46A3 is required to transport catabolites of noncleavable antibody maytansine conjugates from the lysosome to the cytoplasm. Cancer Res..

[127] Sauveur J., Matera E.L., Chettab K., Valet P., Guitton J., Savina A., Dumontet C. (2018). Esophageal cancer cells resistant to T-DM1 display alterations in cell adhesion and the prostaglandin pathway. Oncotarget.

[128] Rasheed Z.A., Rubin E.H. (2003). Mechanisms of resistance to topoisomerase I-targeting drugs. Oncogene.

[129] Mao S., Chaerkady R., Yu W., D’Angelo G., Garcia A., Chen H., Barrett A.M., Phipps S., Fleming R., Hess S. (2021). Resistance to pyrrolobenzodiazepine dimers is associated with SLFN11 downregulation and can be reversed through inhibition of ATR. Mol. Cancer Ther..

[130] Chen Y.F., Xu Y.Y., Shao Z.M., Yu K.D. (2023). Resistance to antibody-drug conjugates in breast cancer: Mechanisms and solutions. Cancer Commun..

[131] Li S., Zhao X., Fu K., Zhu S., Pan C., Yang C., Wang F., To K.K.W., Fu L. (2025). Resistance to antibody-drug conjugates: A review. Acta Pharm. Sin. B.

[132] Musacchio A., Salmon E.D. (2007). The spindle-assembly checkpoint in space and time. Nat. Rev. Mol. Cell Biol..

[133] Dufies M., Verbiest A., Cooley L.S., Ndiaye P.D., He X., Nottet N., Souleyreau W., Hagege A., Torrino S., Parola J. (2021). Plk1, upregulated by HIF-2, mediates metastasis and drug resistance of clear cell renal cell carcinoma. Commun. Biol..

[134] Brito D.A., Rieder C.L. (2006). Mitotic checkpoint slippage in humans occurs via cyclin B destruction in the presence of an active checkpoint. Curr. Biol..

[135] Haag P., Viktorsson K., Lindberg M.L., Kanter L., Lewensohn R., Stenke L. (2009). Deficient activation of Bak and Bax confers resistance to gemtuzumab ozogamicin-induced apoptotic cell death in AML. Exp. Hematol..

[136] Narayan M., Wilken J.A., Harris L.N., Baron A.T., Kimbler K.D., Maihle N.J. (2009). Trastuzumab-induced HER reprogramming in “resistant” breast carcinoma cells. Cancer Res..

[137] Wilson T.R., Fridlyand J., Yan Y., Penuel E., Burton L., Chan E., Peng J., Lin E., Wang Y., Sosman J. (2012). Widespread potential for growth-factor-driven resistance to anticancer kinase inhibitors. Nature.

[138] Saleh R., Elkord E. (2020). Acquired resistance to cancer immunotherapy: Role of tumor-mediated immunosuppression. Semin. Cancer Biol..

[139] Rios-Doria J., Harper J., Rothstein R., Wetzel L., Chesebrough J., Marrero A., Chen C., Strout P., Mulgrew K., McGlinchey K. (2017). Antibody-drug conjugates bearing pyrrolobenzodiazepine or tubulysin payloads are immunomodulatory and synergize with multiple immunotherapies. Cancer Res..

[140] Niu Y., Zhu S., Fu K., Lai Y., Pan C., Yang Y., Li S., Wang X., To K.K.W., Wang F. (2026). Immunogenic cell death as a cornerstone for combination therapies with immune checkpoint blockade. Mol. Cancer.

[141] Nilsson M.B., Le X., Poteete A., Yu X., He J., Huang Q., Shibata Y., Liu X., Moran C., Alizadeh A.A. (2026). Loss of payload sensitivity and other mechanisms of resistance to T-DXd in HER2-mutant NSCLC: Implications for subsequent responsiveness to HER2 TKIs. J. Thorac. Oncol..

[142] Zullo L., Bortolot M., Ogliari F.R., Saw S.P.L., van Geel R., Ter Heine R., Sadowska A., Remon J., Hendriks L. (2026). Antibody-drug conjugates in non-small cell lung cancer. Drugs.

[143] Modi S., Jacot W., Yamashita T., Sohn J., Vidal M., Tokunaga E., Tsurutani J., Ueno N.T., Prat A., Chae Y.S. (2022). Trastuzumab deruxtecan in previously treated HER2-low advanced breast cancer. N. Engl. J. Med..

[144] Li M., Jin M., Peng H., Wang H., Shen Q., Zhang L. (2024). Current status and future prospects of TROP-2 ADCs in lung cancer treatment. Drug Des. Devel. Ther..

[145] Baxi V., Edwards R., Montalto M., Saha S. (2022). Digital pathology and artificial intelligence in translational medicine and clinical practice. Mod. Pathol..

[146] L’Imperio V., Capitoli G., Cazzaniga G., Mannino M., Bono F., Seminati D., Eloy C., Pinto J., Guerini Rocco E., Fassan M. (2025). The routine use of a digital tool for the tumor cell fraction quantification in molecular pathology: An international validation of QuANTUM. Pathologica.

[147] Li J., Jia Z., Dong L., Cao H., Huang Y., Xu H., Xie Z., Jiang Y., Wang X., Liu J. (2024). DNA damage response in breast cancer and its significant role in guiding novel precise therapies. Biomark. Res..

[148] Valle I., Grinda T., Antonuzzo L., Pistilli B. (2025). Antibody-drug conjugates in breast cancer: Mechanisms of resistance and future therapeutic perspectives. npj Breast Cancer.

[149] Fadda G.M., Lobrano R., Casula M., Pisano M., Pazzola A., Cossu A., Palmieri G., Paliogiannis P. (2022). Liquid biopsy in the oncological management of a histologically undiagnosed lung carcinoma: A case report. J. Pers. Med..

[150] Paliogiannis P., Attene F., Cossu A., Defraia E., Porcu G., Carta A., Sotgiu M.I., Pazzola A., Cordero L., Capelli F. (2015). Impact of tissue type and content of neoplastic cells of samples on the quality of epidermal growth factor receptor mutation analysis among patients with lung adenocarcinoma. Mol. Med. Rep..

[151] Bazan Russo T.D., Pepe F., Gristina V., Gottardo A., Russo G., Scimone C., Palumbo L., Busuito G., Incorvaia L., Guerry J.A. (2025). Recent advances in liquid biopsy for precision oncology: Emerging biomarkers and clinical applications in lung cancer. Future Oncol..

[152] Gristina V., Pepe F., Ogliari F.R., Bazan Russo T.D., Gottardo A., Russo G., Incorvaia L., Guerry J.A., Pisapia P., Scimone C. (2025). Smaller, cheaper, faster: Where next for liquid biopsies?. Expert Rev. Mol. Diagn..

[153] Scimone C., Palumbo L., Borea R., Sarracino C., Tomaiuolo I., Di Giovanni D., Alfano S., Nacchio M., Russo G., Russo A. (2025). Innovation in next-generation sequencing in non-small cell lung cancer diagnostics. Expert Rev. Anticancer Ther..

[154] Casula M., Pisano M., Paliogiannis P., Colombino M., Sini M.C., Zinellu A., Santeufemia D., Manca A., Casula S., Tore S. (2023). Comparison between three different techniques for the detection of EGFR mutations in liquid biopsies of patients with advanced stage lung adenocarcinoma. Int. J. Mol. Sci..

[155] Jantus-Lewintre E., Calabuig-Fariñas S., Gandara D., Raez L., Leighl N.B., Fusco N., Khorshid O., Mayo de Las Casas C., Reduzzi C., Urum Y. (2026). Harmonizing definitions in liquid biopsy: A terminology framework by the international society of liquid biopsy. J. Liq. Biopsy.

[156] Boxer E., Feigin N., Tschernichovsky R., Darnell N.G., Greenwald A.R., Hoefflin R., Kovarsky D., Simkin D., Turgeman S., Zhang L. (2025). Emerging clinical applications of single-cell RNA sequencing in oncology. Nat. Rev. Clin. Oncol..

[157] Cheng X., Peng T., Chu T., Yang Y., Liu J., Gao Q., Cao C., Wei J. (2025). Application of single-cell and spatial omics in deciphering cellular hallmarks of cancer drug response and resistance. J. Hematol. Oncol..

[158] See J.E., Barlow S., Arjumand W., DuBose H., Segato Dezem F., Plummer J. (2025). Spatial omics: Applications and utility in profiling the tumor microenvironment. Cancer Metastasis Rev..

[159] Izzo D., Ascione L., Guidi L., Marsicano R.M., Koukoutzeli C., Trapani D., Curigliano G. (2025). Innovative payloads for ADCs in cancer treatment: Moving beyond the selective delivery of chemotherapy. Ther. Adv. Med. Oncol..

[160] Ponte J.F., Sun X., Yoder N.C., Fishkin N., Laleau R., Coccia J., Lanieri L., Bogalhas M., Wang L., Wilhelm S. (2016). Understanding how the stability of the thiol-maleimide linkage impacts the pharmacokinetics of lysine-linked antibody-maytansinoid conjugates. Bioconjug. Chem..

[161] Ringaci A., Grinstaff M.W. (2026). Peptide tag-nology for preparation of site-specific antibody-drug conjugates. Bioconjug. Chem..

[162] Yu X., Ke X., Tang Y., Tang T., Ni Y., Guo L., Cui Y., Mei Y., Xu G., Wu G. (2026). Advancements in dual-load antibody-drug conjugates and challenges with quality analysis. Pharmaceuticals.

[163] Zhou K., Liu X., Zhu H. (2025). Overcoming resistance to antibody-drug conjugates: From mechanistic insights to cutting-edge strategies. J. Hematol. Oncol..

[164] Gerber H.P., Sapra P., Loganzo F., May C. (2016). Combining antibody-drug conjugates and immune-mediated cancer therapy: What to expect?. Biochem. Pharmacol..

[165] Le X., Kim T.M., Loong H.H., Prelaj A., Goh B.C., Li L., Fang Y., Lu S., Dong X., Wu L. (2025). Sevabertinib in advanced HER2-mutant non-small-cell lung cancer. N. Engl. J. Med..

[166] Heymach J.V., Ruiter G., Ahn M.J., Girard N., Smit E.F., Planchard D., Wu Y.L., Cho B.C., Yamamoto N., Sabari J.K. (2025). Zongertinib in previously treated HER2-mutant non-small-cell lung cancer. N. Engl. J. Med..

[167] Chen Y., Zhang Y., Tian C., Li Z., Guo G. (2026). Evolution and recent advances in antibody-drug conjugate therapy for lung cancer: A comprehensive systematic review based on the Trialtrove database (inception to July 1, 2025). Front. Oncol..

[168] Lin H., Ma X., Zhu X., Zhong L. (2025). The potential of antibody-drug conjugates in immunotherapy for non-small cell lung cancer: Current progress and future. Front. Oncol..

